# Supplementary material for: Clinical recognition of frontotemporal dementia with right temporal predominance: a consensus statement from the International Working Group
Source: Commun Med (Lond). 2025 Dec 12;5:523. doi: 10.1038/s43856-025-01252-4 (PMC12700944; doi:10.1038/s43856-025-01252-4)

SUPPLEMENTARY FIGURES

Supplementary Figure 1: PRISMA flow diagram for the systematic review on knowledge for people
PRISMA flow diagram for the systematic review on knowledge for people. The diagram summarizes the number of records identified through database searches (PubMed, Embase), duplicates removed, records screened, full-text articles assessed for eligibility, and studies included in the final analysis. RATL, right anterior temporal lobe.

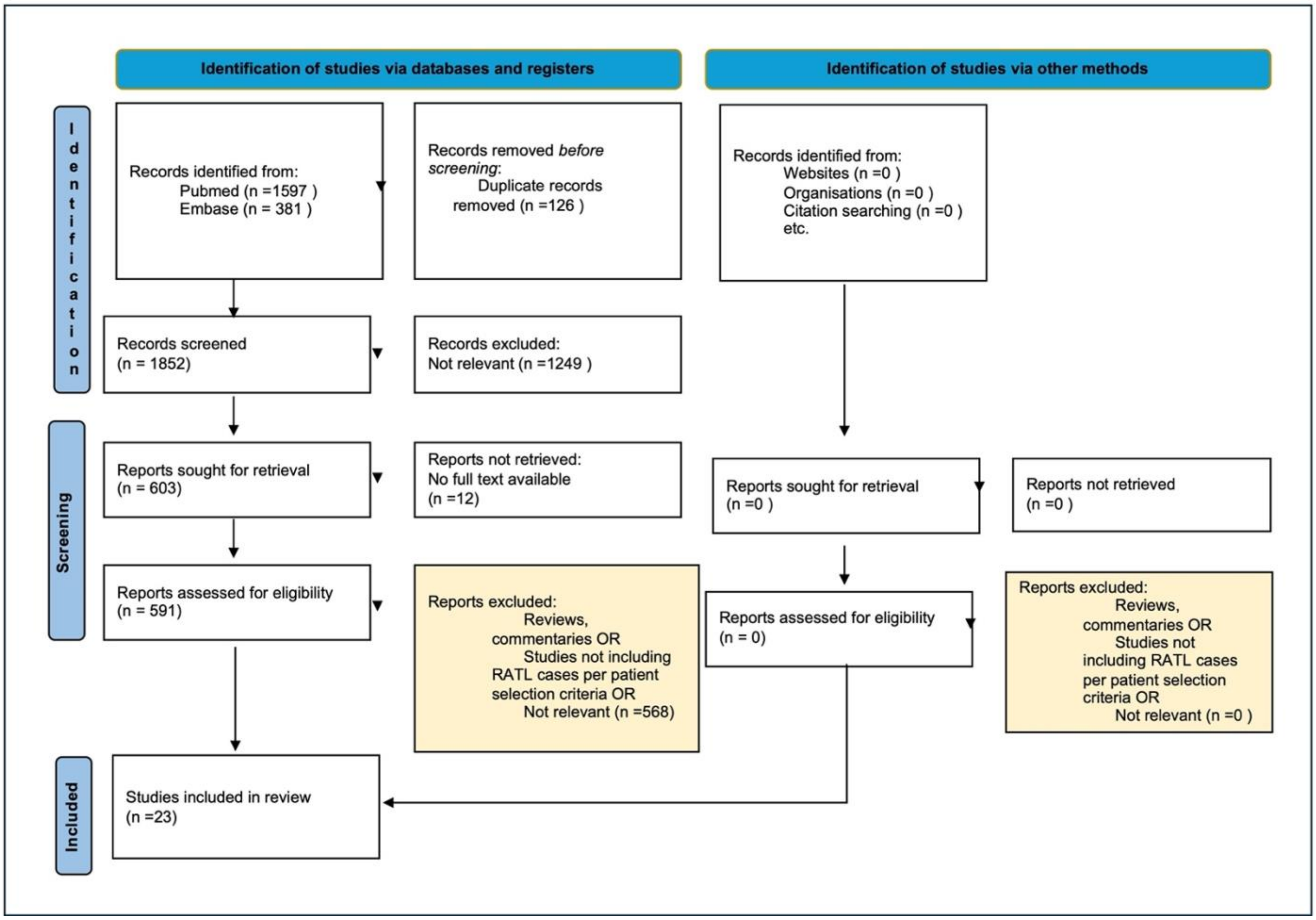

Supplementary Figure 2: PRISMA flow diagram for the systematic review on knowledge for emotions
PRISMA flow diagram for the systematic review on knowledge for emotions. The diagram summarizes the number of records identified through database searches (PubMed, Embase), duplicates removed, records screened, full-text articles assessed for eligibility, and studies included in the final analysis. RATL, right anterior temporal lobe.

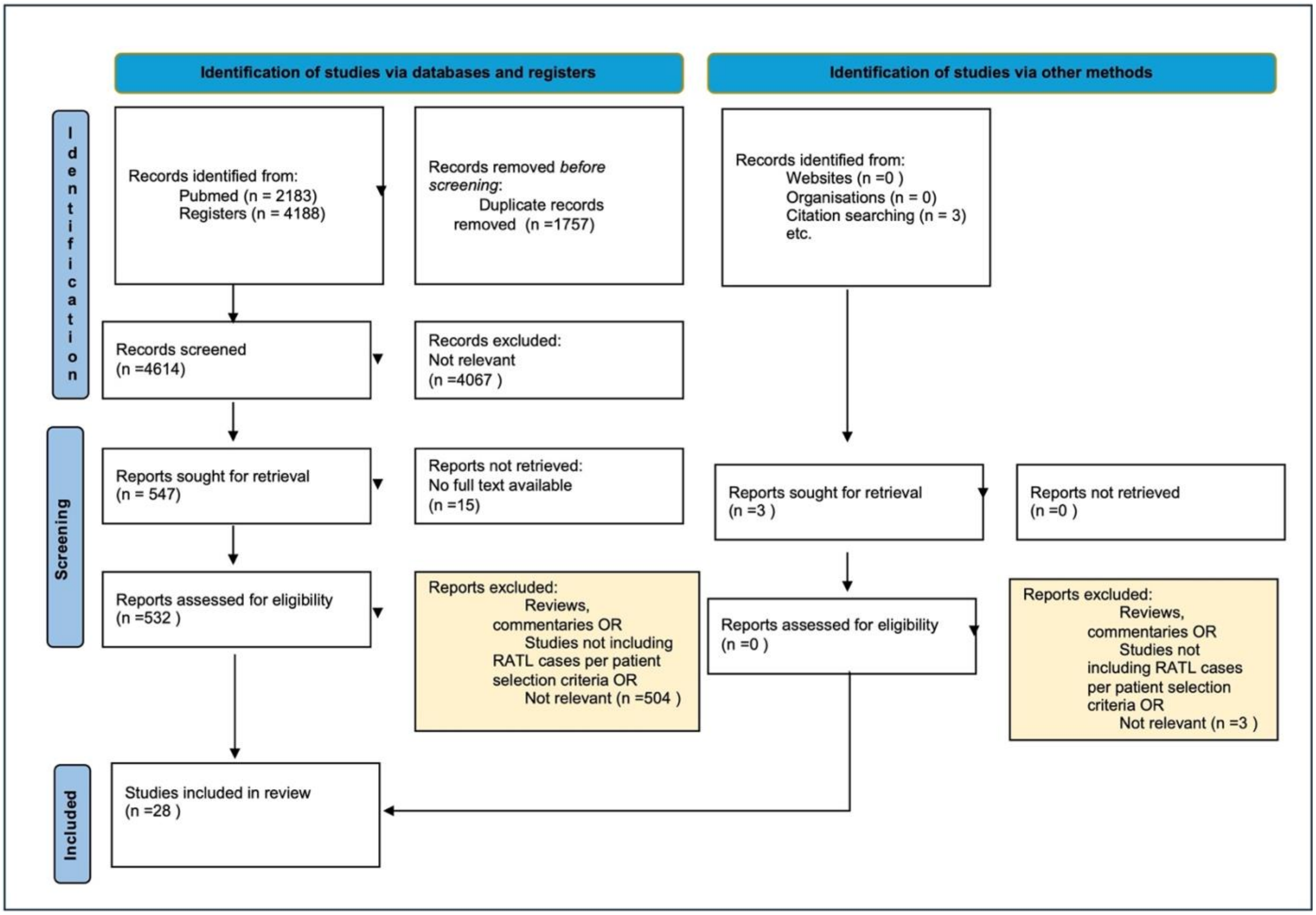

Supplementary Figure 3: PRISMA flow diagram for the systematic review on knowledge for social interactions

PRISMA flow diagram for the systematic review on knowledge for social interactions. The diagram summarizes the number of records identified through database searches (PubMed, Embase), duplicates removed, records screened, full-text articles assessed for eligibility, and studies included in the final analysis. RATL, right anterior temporal lobe.

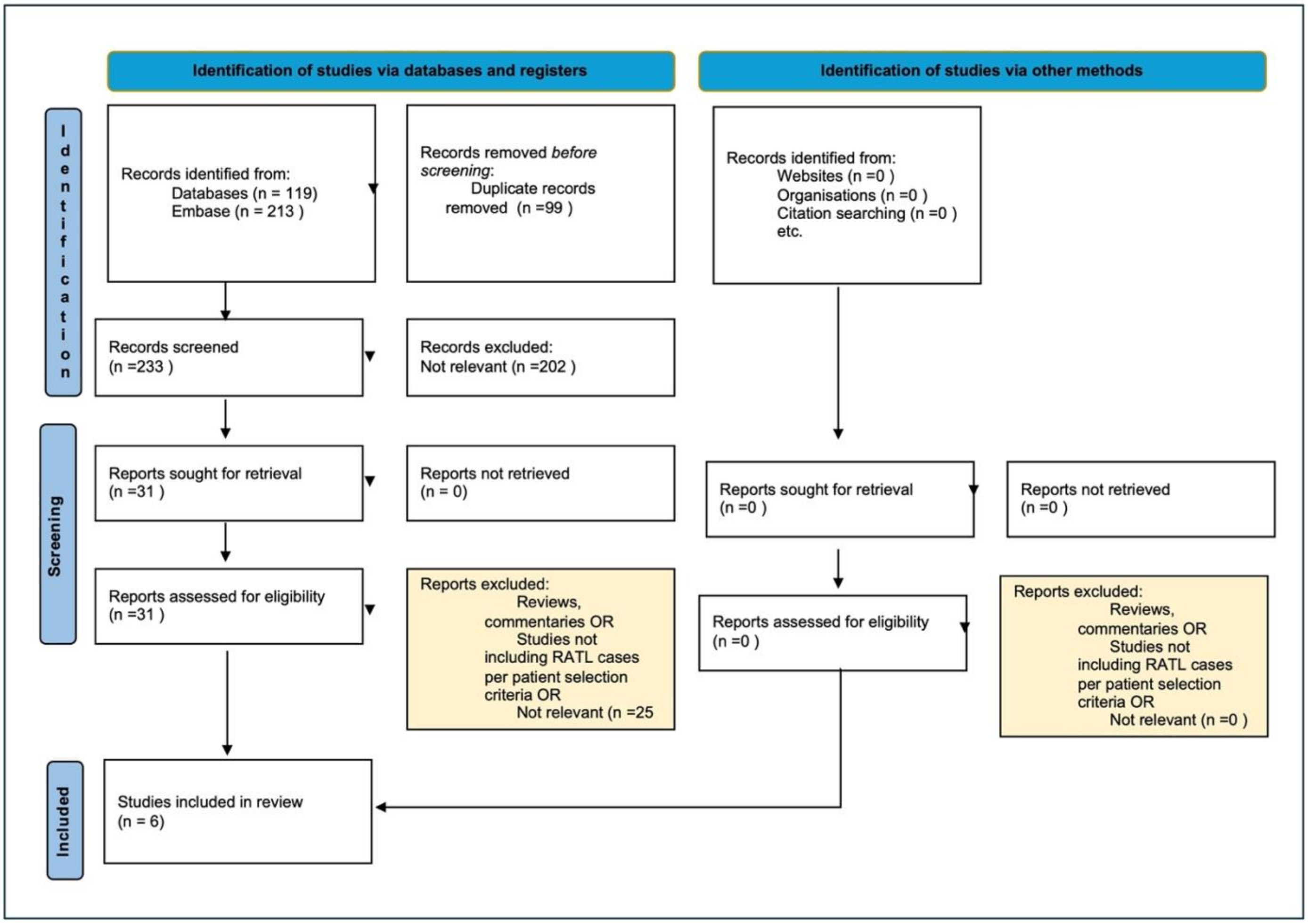

Supplementary Figure 4: PRISMA flow diagram for the systematic review on knowledge for tastes
PRISMA flow diagram for the systematic review on knowledge for tastes. The diagram summarizes the number of records identified through database searches (PubMed, Embase), duplicates removed, records screened, full-text articles assessed for eligibility, and studies included in the final analysis. RATL, right anterior temporal lobe.

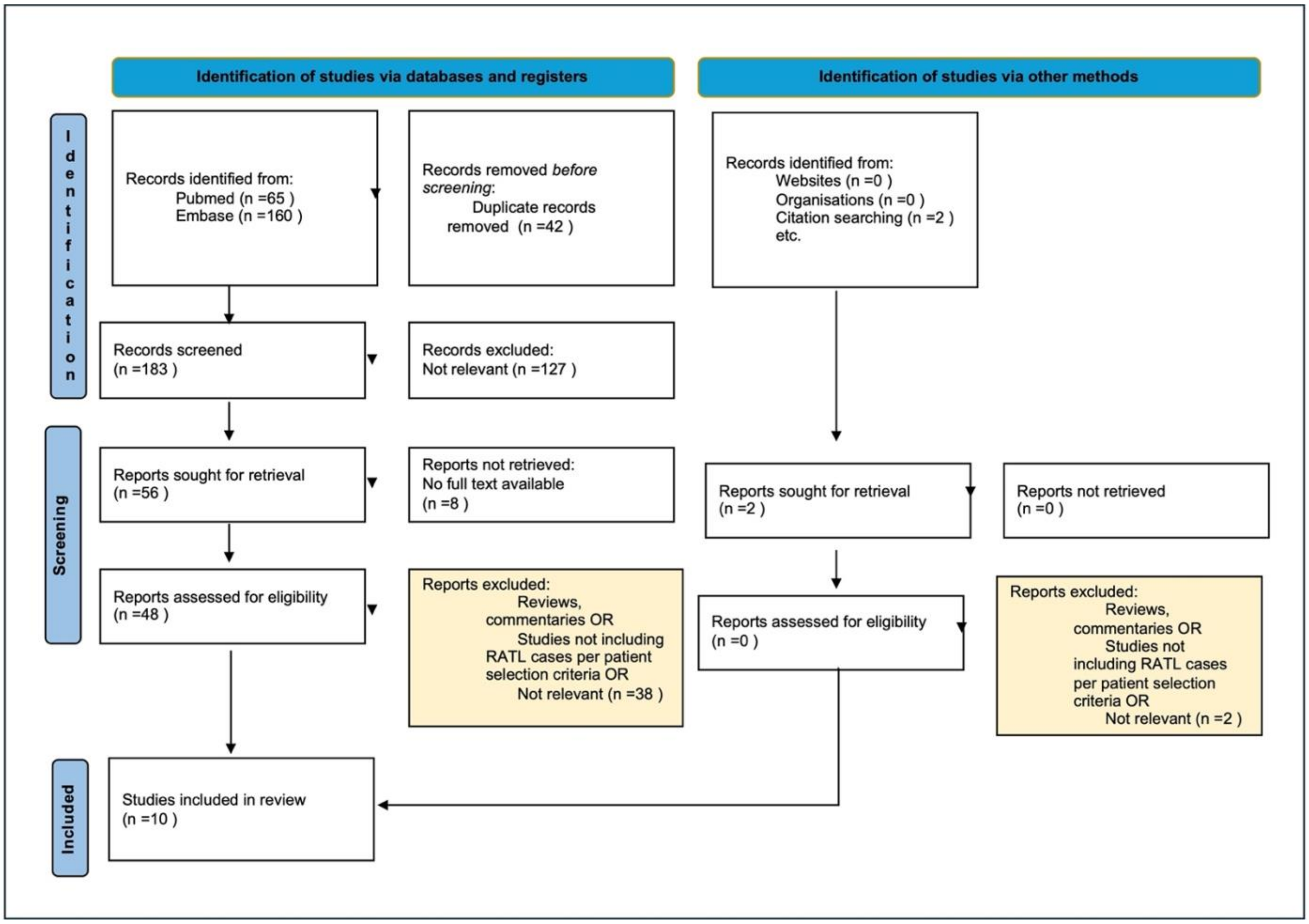

Supplementary Figure 5: PRISMA flow diagram for the systematic review on knowledge for sounds
PRISMA flow diagram for the systematic review on knowledge for sounds. The diagram summarizes the number of records identified through database searches (PubMed, Embase), duplicates removed, records screened, full-text articles assessed for eligibility, and studies included in the final analysis. RATL, right anterior temporal lobe.

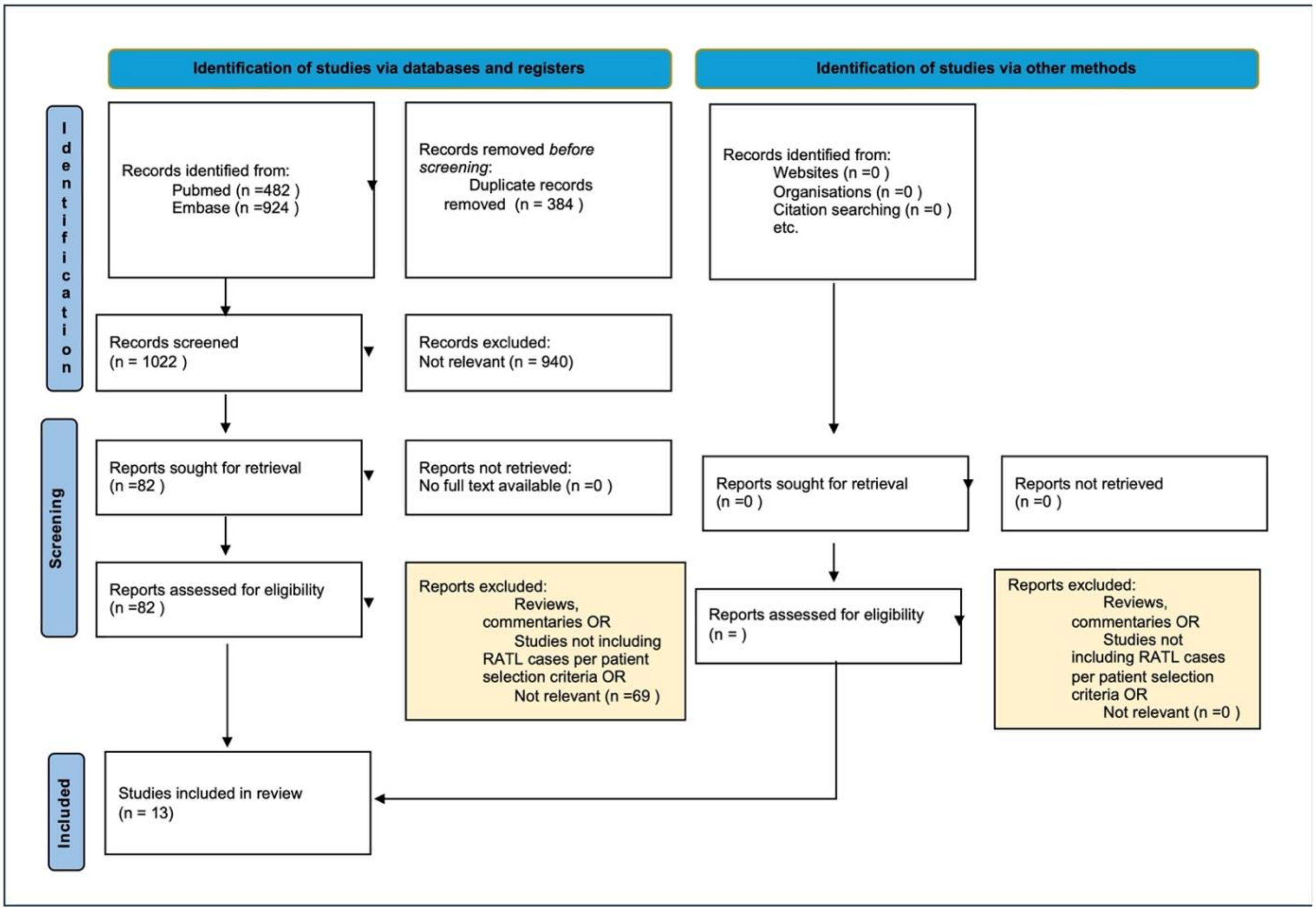

Supplementary Figure 6: PRISMA flow diagram for the systematic review on knowledge for smells
PRISMA flow diagram for the systematic review on knowledge for smells. The diagram summarizes the number of records identified through database searches (PubMed, Embase), duplicates removed, records screened, full-text articles assessed for eligibility, and studies included in the final analysis. RATL, right anterior temporal lobe.

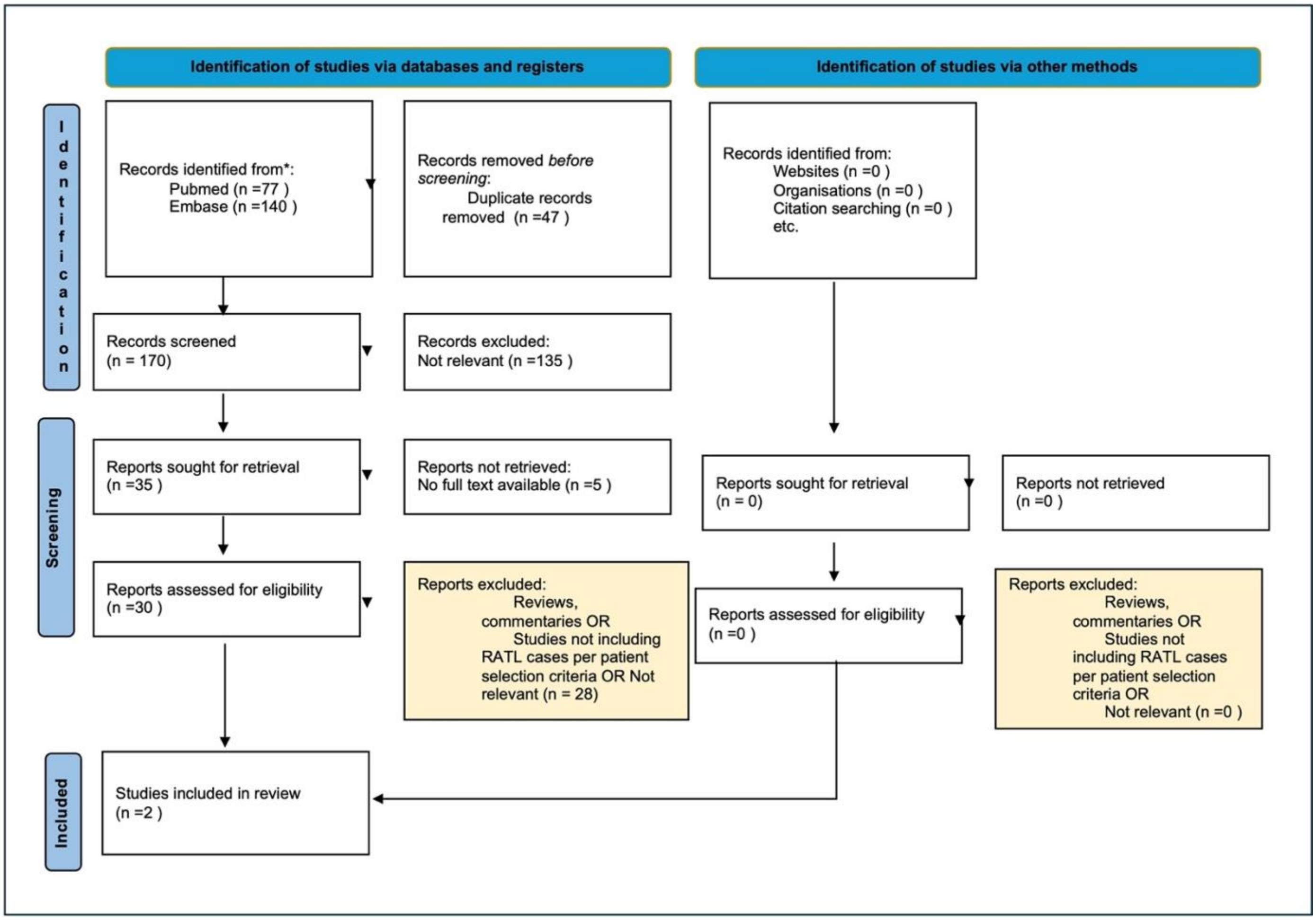

Supplementary Figure 7: PRISMA flow diagram for the systematic review on knowledge for landmarks

PRISMA flow diagram for the systematic review on knowledge for landmarks. The diagram summarizes the number of records identified through database searches (PubMed, Embase), duplicates removed, records screened, full-text articles assessed for eligibility, and studies included in the final analysis. RATL, right anterior temporal lobe.

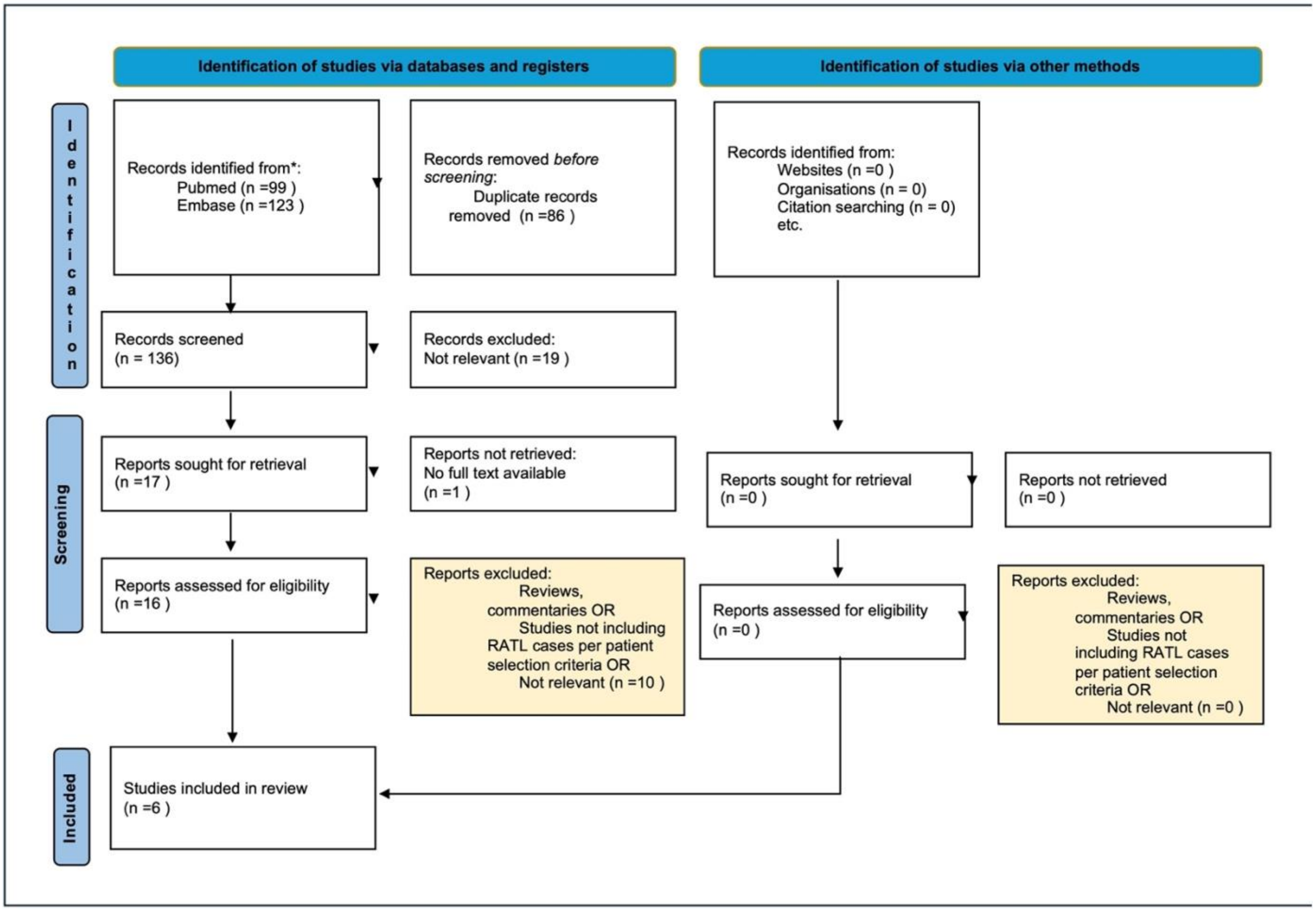

Supplementary Figure 8: PRISMA flow diagram for the systematic review on knowledge for bodily sensations

PRISMA flow diagram for the systematic review on knowledge for bodily sensations. The diagram summarizes the number of records identified through database searches (PubMed, Embase), duplicates removed, records screened, full-text articles assessed for eligibility, and studies included in the final analysis. RATL, right anterior temporal lobe.

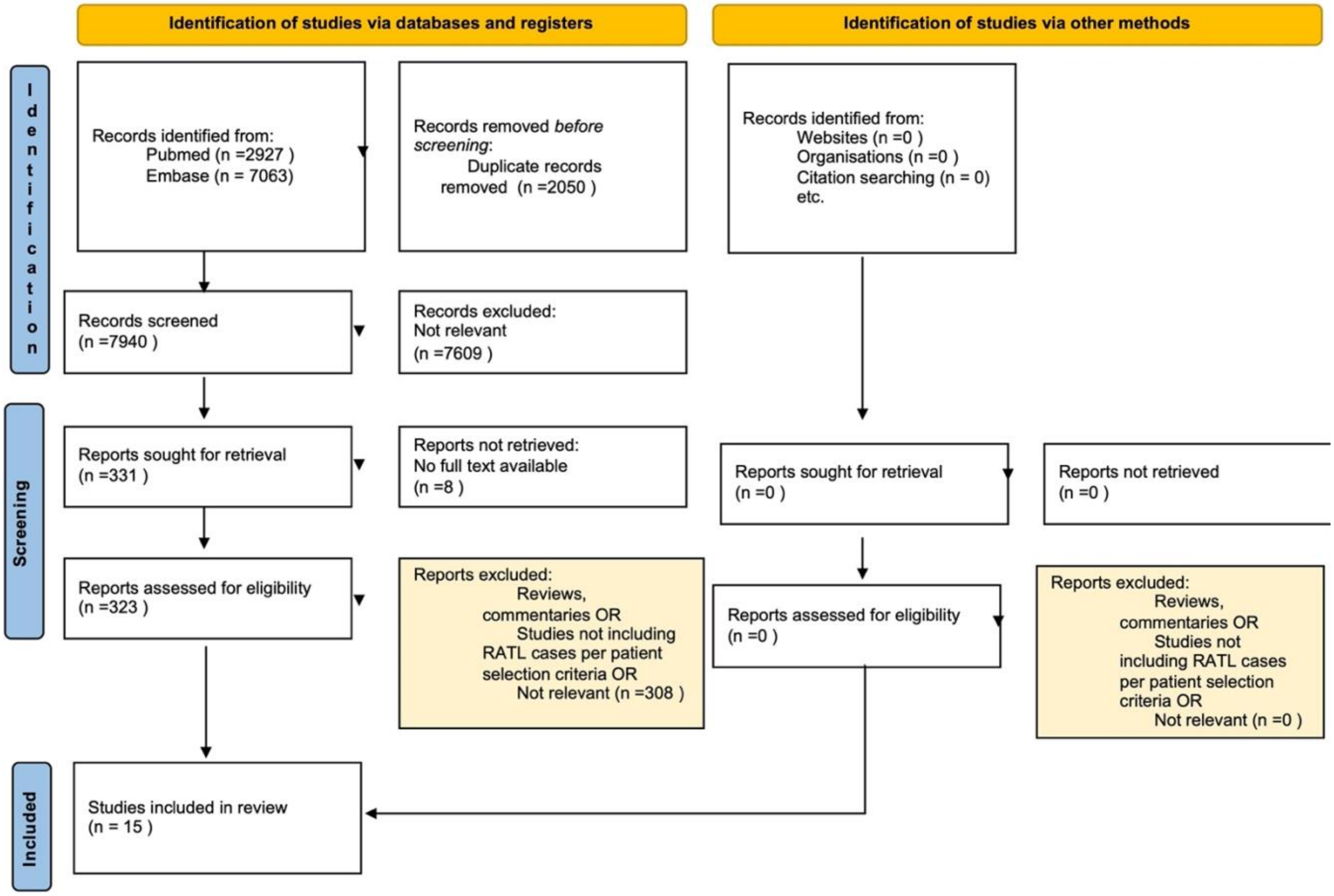

Supplementary Figure 9: PRISMA flow diagram for the systematic review on knowledge for visual stimuli

PRISMA flow diagram for the systematic review on knowledge for visual stimuli. The diagram summarizes the number of records identified through database searches (PubMed, Embase), duplicates removed, records screened, full-text articles assessed for eligibility, and studies included in the final analysis. RATL, right anterior temporal lobe.

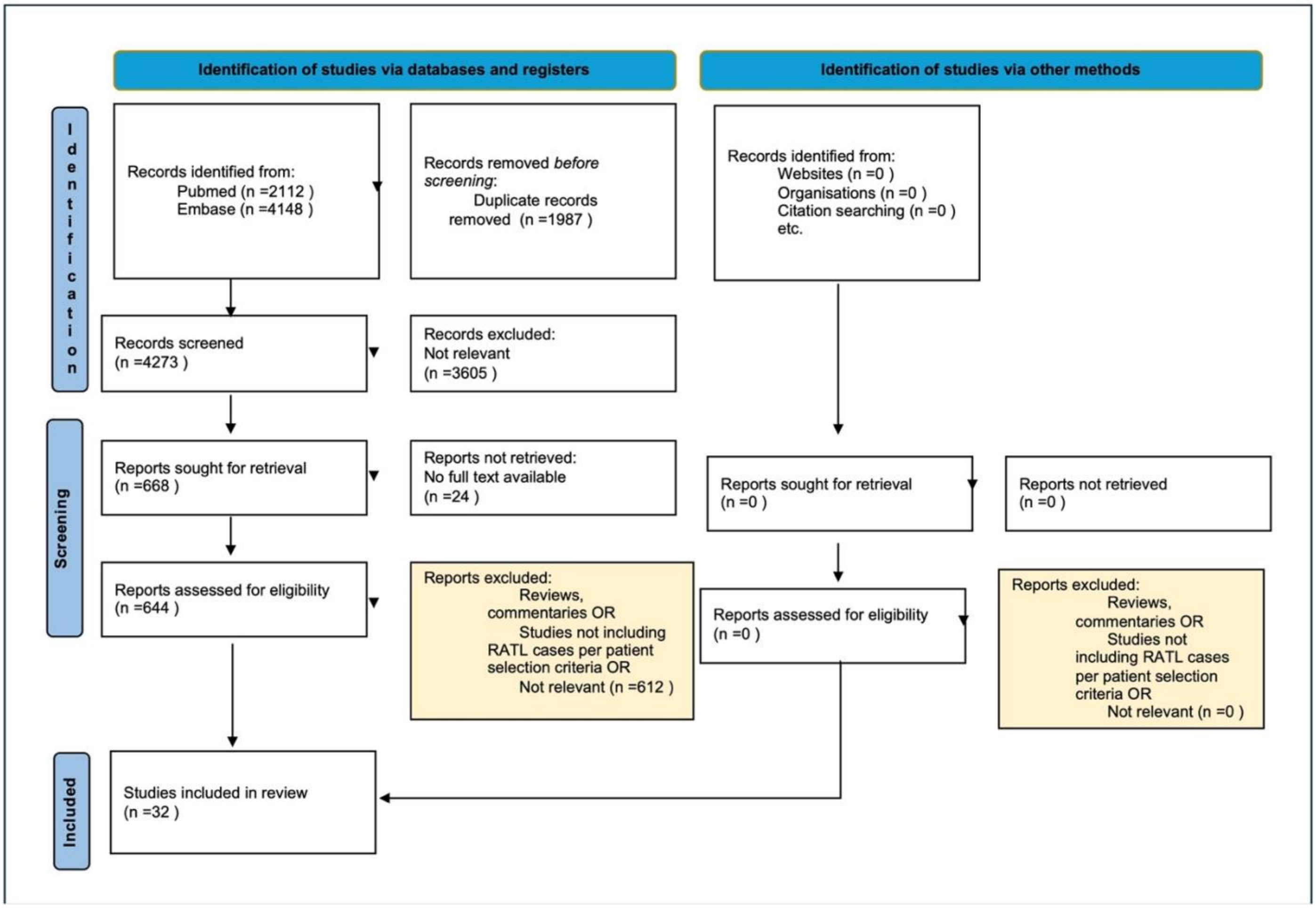

Supplementary Figure 10: PRISMA flow diagram for the systematic review on memory deficit
PRISMA flow diagram for the systematic review on memory deficit. The diagram summarizes the number of records identified through database searches (PubMed, Embase), duplicates removed, records screened, full-text articles assessed for eligibility, and studies included in the final analysis. RATL, right anterior temporal lobe.

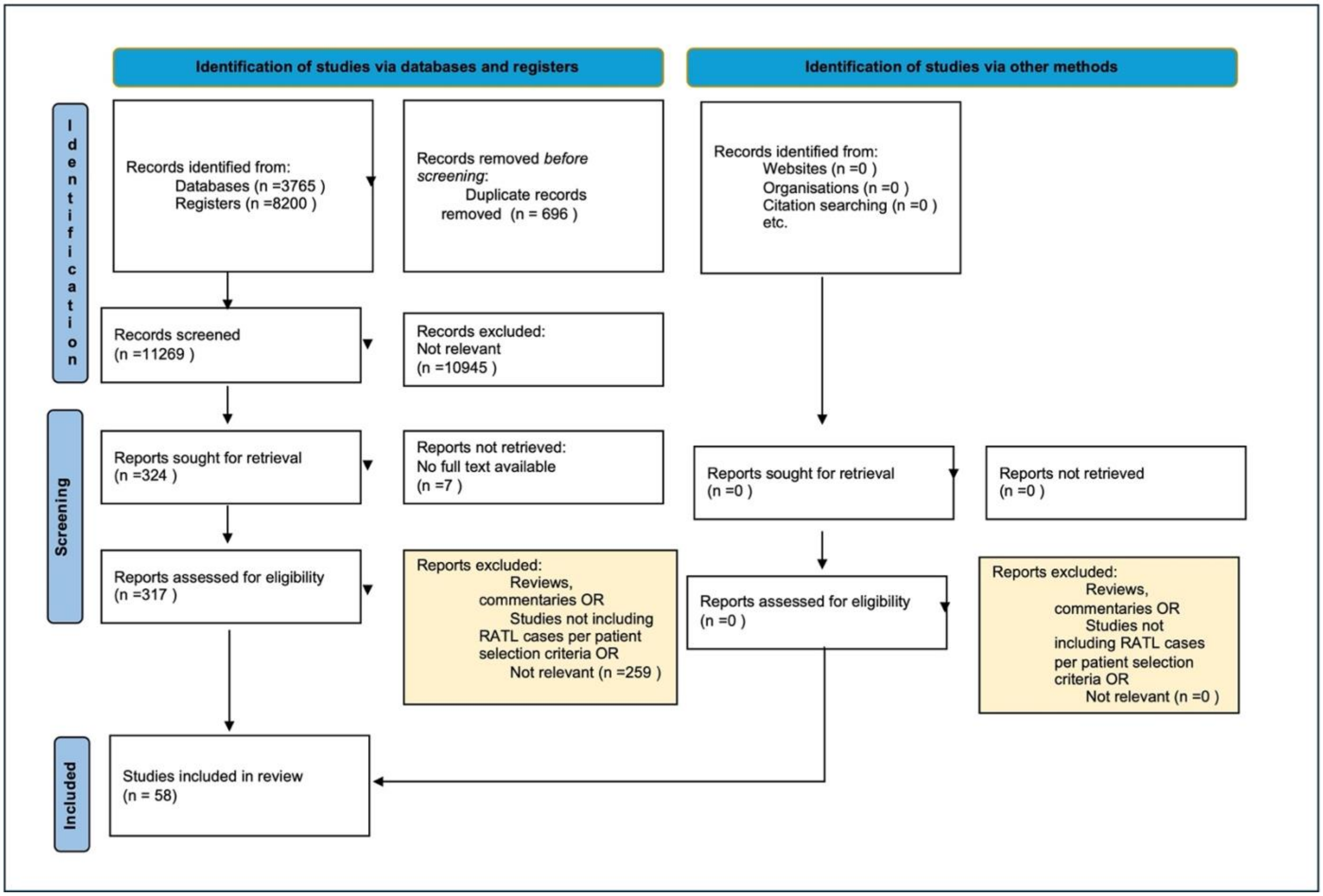

Supplementary Figure 11: PRISMA flow diagram for the systematic review on apathy

PRISMA flow diagram for the systematic review on apathy. The diagram summarizes the number of records identified through database searches (PubMed, Embase), duplicates removed, records screened, full-text articles assessed for eligibility, and studies included in the final analysis. RATL, right anterior temporal lobe.

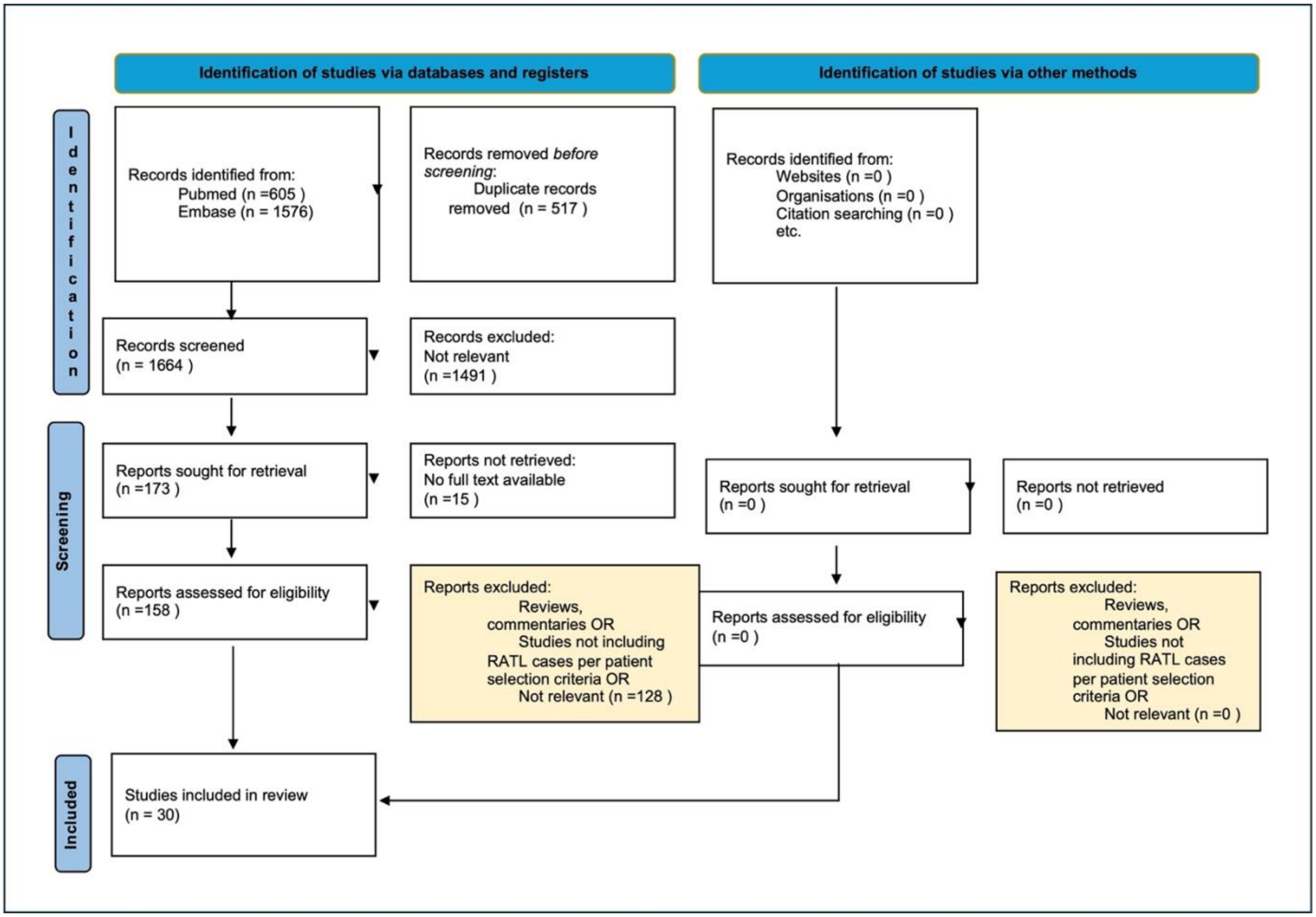

Supplementary Figure 12: PRISMA flow diagram for the systematic review on rigid behavior
PRISMA flow diagram for the systematic review on rigid behavior. The diagram summarizes the number of records identified through database searches (PubMed, Embase), duplicates removed, records screened, full-text articles assessed for eligibility, and studies included in the final analysis. RATL, right anterior temporal lobe.

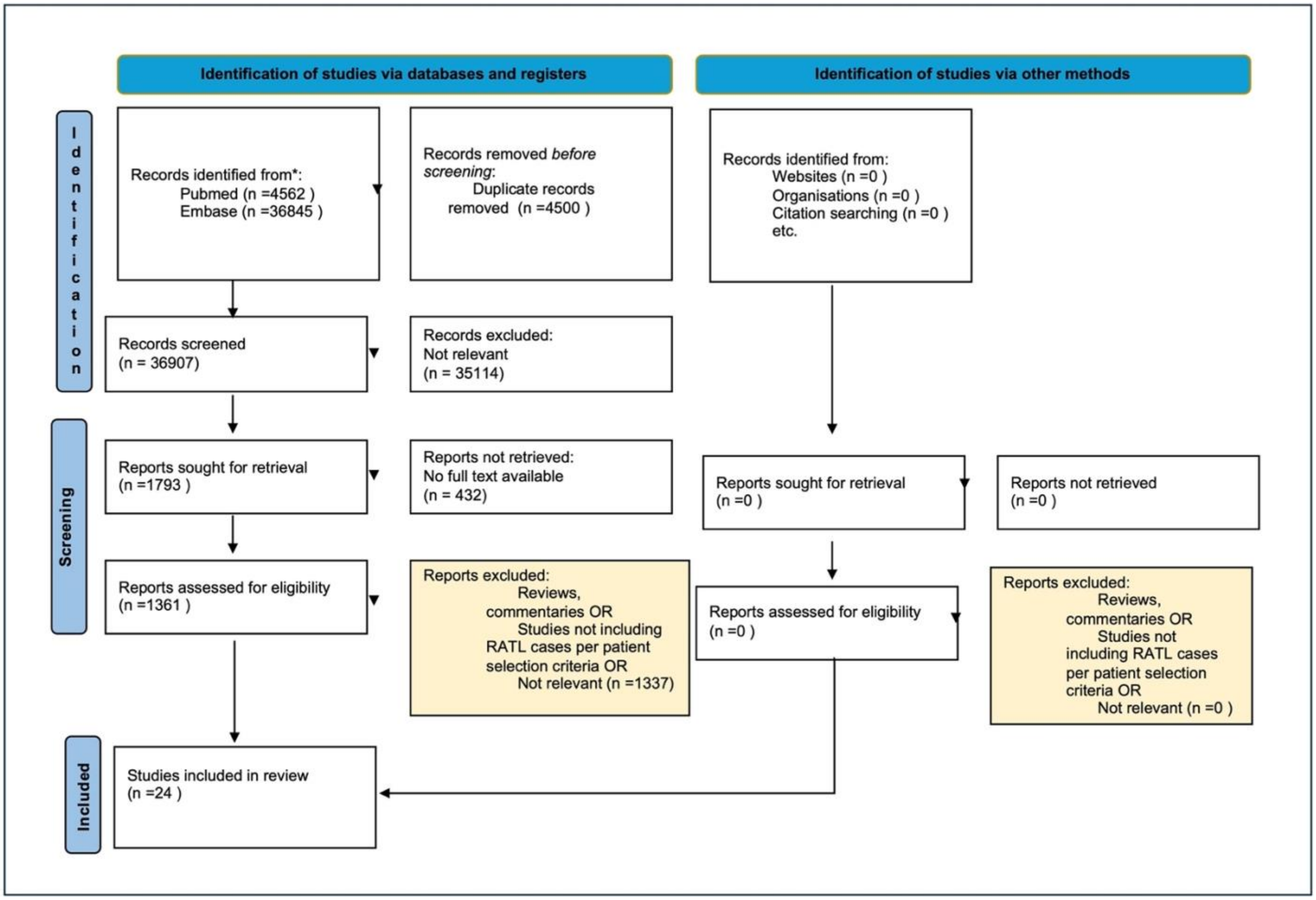

Supplementary Figure 13: PRISMA flow diagram for the systematic review on psychiatric symptoms
PRISMA flow diagram for the systematic review on psychiatric symptoms. The diagram summarizes the number of records identified through database searches (PubMed, Embase), duplicates removed, records screened, full-text articles assessed for eligibility, and studies included in the final analysis. RATL, right anterior temporal lobe.

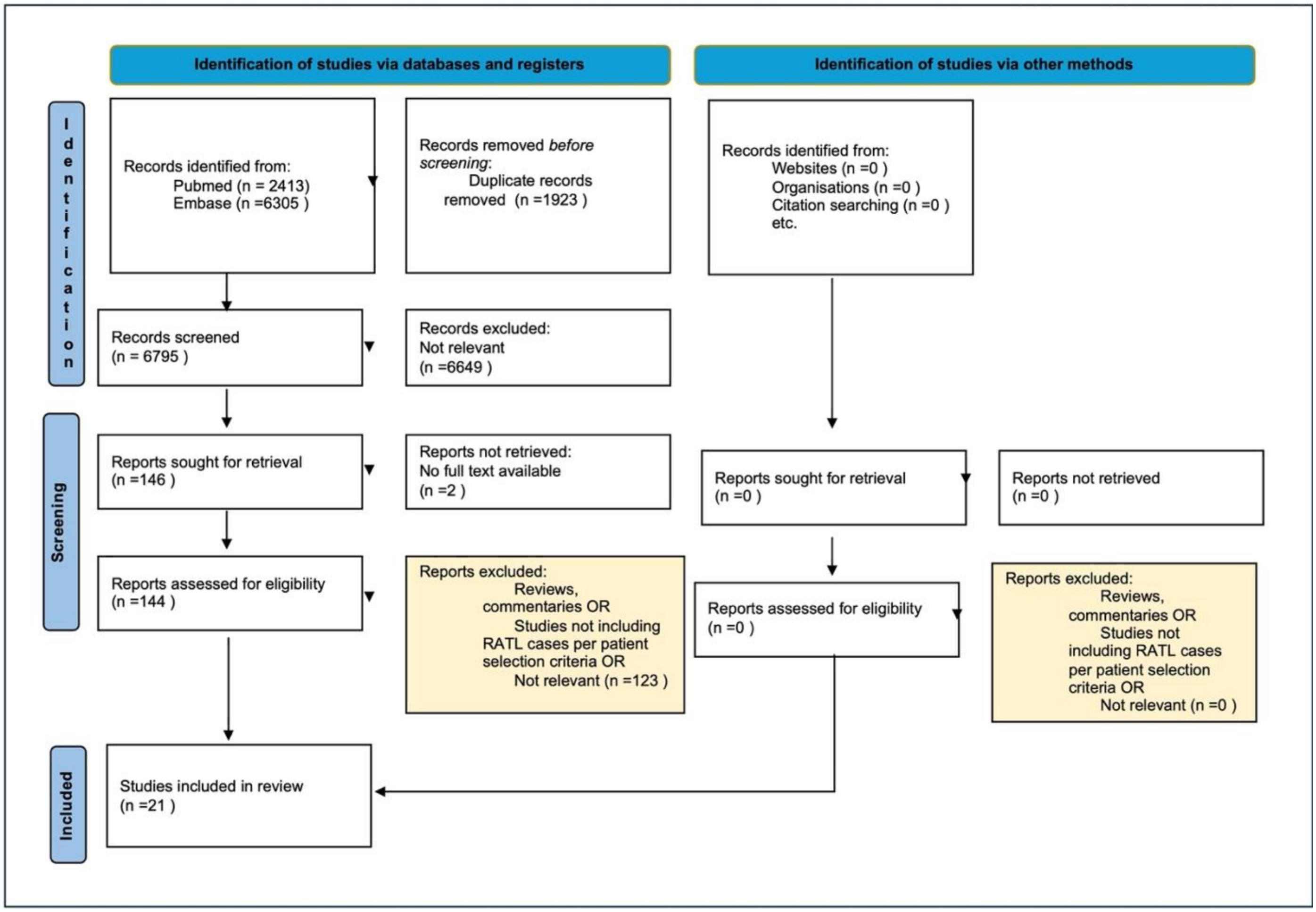

Supplement: Supplementary file 1 — Supplementary Figs. [file 43856_2025_1252_MOESM1_ESM.pdf]
