# Supplementary material for: Clinical recognition of frontotemporal dementia with right temporal predominance: a consensus statement from the International Working Group
Source: Commun Med (Lond). 2025 Dec 12;5:523. doi: 10.1038/s43856-025-01252-4 (PMC12700944; doi:10.1038/s43856-025-01252-4)
Supplement: Supplementary file 2 — Description of Additional Supplementary Files [file 43856_2025_1252_MOESM2_ESM.docx]

**Description of Additional Supplementary Files**

File name: Supplementary Data File 1

Description: Contains Supplementary Table 1, which lists the search terms used in the systematic review.

File name: Supplementary Data File 2

Description: Includes Supplementary Table 2, which summarizes the search results.

File name: Supplementary Data File 3

Description: Includes Supplementary Table 3, which provides the final list of all studies included in the systematic review.

File name: Supplementary Data File 4

Description: Includes Supplementary Table 4, which provides interpretations of the reported symptoms and consensus recommendations.

File name: Supplementary Figures

Description: Includes Supplementary Figures 1-13, and their titles and legends
