# Supplementary material for: Clinical recognition of frontotemporal dementia with right temporal predominance: a consensus statement from the International Working Group
Source: Commun Med (Lond). 2025 Dec 12;5:523. doi: 10.1038/s43856-025-01252-4 (PMC12700944; doi:10.1038/s43856-025-01252-4)
Supplement: Supplementary file 3 — Supplementary Data File 1 [file 43856_2025_1252_MOESM3_ESM.docx]

**Supplementary Table 1. Search terms**

| “frontotemporal dementia” OR “semantic dementia” OR "semantic variant primary progressive aphasia” OR “behavioral variant frontotemporal dementia” OR “temporal variant frontotemporal dementia” OR “frontotemporal lobar degeneration” OR “Pick’s Disease” OR “right temporal” OR “right anterior*” OR “temporal pole” | AND | “prosopagnosia” OR “associative prosopagnosia” OR “face recognition deficit” OR “person knowledge” OR “semantics for people” OR “face agnosia” OR “face blindness” OR “person identification” OR “person-specific” OR “person recognition” OR “face recognition” |
| --- | --- | --- |
|  |  | “emotion recognition” OR “emotion labeling” OR “emotion naming” OR “emotion discrimination” OR “alexithymia” OR “emotion reading” OR “emotional semantic” OR “emotion understanding” OR “emotion perception” OR “empathy” OR “lack of empathy” OR “loss of empathy” OR “emotion” OR “feeling” OR “theory of mind” OR “perspective taking” OR “mentalizing” OR “emotional caricatures” OR “misplaced empathy” OR “affect” OR “affective” |
|  |  | “social cognition” OR “social semantic” OR “paralinguistic” OR “social knowledge” OR “disinhibition” OR “socially inappropriate” OR “social norms” OR “decision making” OR “moral dilemma” OR “social behaviour” OR “social behavior” “impulsivity” “judgment” OR “lack of judgment” OR “criminal behavior” OR “crime” OR “delinquency” OR “misdemeanour” OR “misdemeanor” OR “humor” OR “humour” OR “sarcasm” OR “irony” OR “intertemporal choices” OR “delay discounting” OR “faux pas” |
|  |  | “taste” OR “gustatory agnosia” OR “flavor” OR “flavour” OR “flavor knowledge” OR “taste recognition” OR “taste identification” OR “taste discrimination” OR “food preference” |
|  |  | “sound” OR “vocal agnosia” OR “voice agnosia” OR “phonagnosia” OR “audio” OR “sound knowledge” OR “sound recognition” OR “voice recognition” OR “sound identification” OR “sound discrimination” OR “prosody” OR “tone” OR “auditory agnosia” |
|  |  | “smell” OR “odor” OR “odour” OR “olfactory agnosia” OR “smell knowledge” OR “smell recognition” OR “smell identification” OR “smell discrimination” |
|  |  | “visceral” OR “visceral agnosia” OR “interoceptive agnosia” OR “somatic” OR “psychosomatic” OR “hysteria” OR “pain” OR “temperature” OR “headache” OR “hypochondria” OR “hypochondriacal preoccupation” OR “somatoform” OR “somatoform symptoms” OR “somatoform disorder” OR “hyperalgesia” OR “itching” OR “asomatognosia” OR “alexisomia” OR “bodily sensations” OR “bodily sensation recognition” OR “bodily sensation identification” OR “bodily sensation discrimination” OR “interoceptive” OR “interoception” OR “thermoception” OR “nociception” OR “autonomic” OR “homeostatic” OR “tactile” OR “haptic” OR “somatosensory” OR “self” OR “non-self” OR “sleep” |
|  |  | “landmarks” OR “topographagnosia” OR “landmarks semantic” OR “recognition of monuments” OR “place knowledge” OR “getting lost” |
|  |  | “visual semantic” OR “visual agnosia” OR “object naming” OR “object identification” OR “object recognition” OR “non-verbal semantic” OR “semantic” |
|  |  | “memory” OR “amnesia” OR “episodic memory” OR “semantic memory” OR “autobiographical memory” OR “visual memory” OR “verbal memory” OR “social memory” OR “emotional memory” |
|  |  | “apathy” OR “motivation” OR “affective apathy” OR “social withdrawal” OR “emotional apathy” OR “social interest” OR “social apathy” OR “inertia” OR “anhedonia” OR “reward processing” OR “reward seeking” OR “abulia” OR “aboulia” OR “loss of motivation” |
|  |  | “mental rigidity” OR “mental inflexibility” OR “rigid thinking” OR “hyper-focused interests” OR “preoccupation” OR “compulsion” OR “compulsive” OR “obsession” OR “specific interest” OR “hedonic valuation” OR “reward” OR “hyper-religiosity” OR “mystic” OR “music*” OR “art*” OR “esthetic” OR “color” OR “puzzle” OR “time” OR “schedule” OR “ritualistic” OR “fixated” OR “ritual” OR “religion” OR “colour” OR “game” OR “indulge” OR “punctuality” OR “clock-watching” OR “fixed” OR “routine” OR “gambling” |
|  |  | “depression” OR “mania” OR “mood” OR “altered emotion” OR “affective dysregulation” OR “psychiatric” OR “delusion” OR “hallucination” OR “hyper” OR “impatient” OR “euphoric” OR “cross-modal” OR “synaesthetic” |
