# Supplementary material for: Clinical recognition of frontotemporal dementia with right temporal predominance: a consensus statement from the International Working Group
Source: Commun Med (Lond). 2025 Dec 12;5:523. doi: 10.1038/s43856-025-01252-4 (PMC12700944; doi:10.1038/s43856-025-01252-4)
Supplement: Supplementary file 4 — Supplementary Data File 2 [file 43856_2025_1252_MOESM4_ESM.docx]

**Supplementary Table 2. Search Results**

| **Category** | **Keywords related disease**  **(Supp Table 1, column 1)** | **Keywords related symptom**  **(Supp Table 1, column 2)** | **Overlap** | **Final Included** |
| --- | --- | --- | --- | --- |
| Person | P: 25,252  E: 50,249 | P: 36,382  E: 7,870 | P: 1,597  E:381 | 23 |
| Emotion | P: 25,252  E: 50,249 | P: 1,073,573  E: 1,416,355 | P: 2,183  E: 4,186 | 28 |
| Social Interaction | P: 25,252  E: 50,249 | P: 85,096  E: 112,956 | P: 119  E: 213 | 6 |
| Taste | P: 25,252  E: 50,249 | P: 70,181  E: 110,265 | P: 65  E: 160 | 10 |
| Sound | P: 25,252  E: 50,249 | P: 222,842  E: 349,233 | P: 482  E: 924 | 13 |
| Smell | P: 25,252  E: 50,249 | P: 46,946  E: 62,991 | P: 77  E: 140 | 2 |
| Landmarks | P: 25,252  E: 50,249 | P: 25,299  E: 30,577 | P: 99  E: 123 | 6 |
| Bodily Sensations | P: 25,252  E: 50,249 | P: 3,614,845  E: 5,139,752 | P: 2,927  E: 7,063 | 15 |
| Visual Stimuli | P: 25,252  E: 50,249 | P: 54,034  E: 68,508 | P:2,112  E: 4,148 | 32 |
| Memory | P: 25,252  E: 50,249 | P: 368,679  E: 541,720 | P: 3,765  E: 8,200 | 58 |
| Apathy | P: 25,252  E: 50,249 | P: 169,814  E: 222,722 | P: 605  E: 1,576 | 30 |
| Rigid Behavior | P: 25,252  E: 50,249 | P: 6,081,465  E: 34,708,791 | P: 4,562  E: 36,708,791 | 24 |
| Psychiatric | P: 25,252  E: 50,249 | P: 894,783  E: 1,429,260 | P: 2,413  E: 6,305 | 21 |

P: Pubmed, E: Embase. Numbers are representing the amount of the articles after each search. Final included: The number of the articles after the exclusion of duplications, irrelevant articles, reviews, metanalyses, commentaries, full text unavailable abstracts, and studies not including Right Anterior Temporal Lobe (RATL) cases based on patient selection criteria.
