# Supplementary material for: Clinical recognition of frontotemporal dementia with right temporal predominance: a consensus statement from the International Working Group
Source: Commun Med (Lond). 2025 Dec 12;5:523. doi: 10.1038/s43856-025-01252-4 (PMC12700944; doi:10.1038/s43856-025-01252-4)
Supplement: Supplementary file 5 — Supplementary Data File 3 [file 43856_2025_1252_MOESM5_ESM.docx]

**Supplementary Table 3. Included studies**

| **Author/ year** | **Description of the syndrome/ Subject selection** | **Sample size** | **Diversity (Ethnicity/ Country)** | **Biomarker** | **FTLD Definite Cases** | **Symptom(s) studied (terminology in the original paper)** | **Measurement of symptoms** |
| --- | --- | --- | --- | --- | --- | --- | --- |
| Tyrrell et al., 1990^1^ \| Progressive degeneration of the right temporal lobe studied with positron emission tomography | Focal right temporal lobe degeneration/ PET hypometabolism | 1 | n.a./UK | n.a. | n.a. | Prosopagnosia | Face perception, famous face identification, and familiarity judgement. |
| Barbarotto et al., 1995^2^ \| Slowly progressive semantic impairment with category specificity | SD with predominant right temporal atrophy/ MRI visual assessment | 1 | n.a./ Italy | n.a. | n.a. | Lexical-semantic deficit that was more severe for living things, famous people and architectural knowledge and concerned both visual and encyclopedic information | Medical notes, semantic questionnaire, naming, pointing, drawing, and a battery for face recognition as well as formal testing for memory, language, attention, executive and visuospatial functions. |
| Evans et al., 1995^3^ \| Progressive prosopagnosia associated with selective right temporal lobe atrophy: A new syndrome? | Right temporal lobe atrophy/ SPECT and visual assessment of MRI | 1 | n.a./UK | n.a. | n.a. | Person-based semantic knowledge deficit  Knowledge for people from names was originally much better than from faces, but clearly declined on follow-up | Face perception, recognition, emotion expression |
| Kazui et al., 1995^4^ \| A case of predominantly right-temporal lobe atrophy with disturbance of identifying familiar faces | Predominant right-temporal lobe atrophy / Visual assessment of atrophy | 1 | n.a./ Japan | n.a. | n.a. | Person (face and voice), famous common buildings and animal identification deficits, and, memory deficit for social events | Tests for face perception, face matching, recognition of age and sex, and meaning of facial expressions, facial identity, faces of families and famous persons and formal assessment for language, episodic memory, executive and visuospatial functions |
| Edwards Lee et al., 1997^5^ \| The temporal variant of frontotemporal dementia | Right sided temporal variant FTD/ Visual atrophy assessment | 5 | n.a./ USA | n.a. | n.a. | Irritability, impulsiveness, bizarre alterations in dress, limited and fixed ideas, decreased facial expression and increased visual alertness | Medical notes, formal assessment for memory, attention, language and executive functions |
| Miller et al., 1998^6^ \| Emergence of artistic talent in frontotemporal dementia | FTD with right temporal atrophy/ Autopsy | 1 | n.a./ USA | n.a. | Non-AD pathology | Social withdraw, emergence of artistic talent, fixed color preference in the paintings. | Clinical notes |
| Gentileschi et al., 1999^7^ \| Progressive Defective Recognition of Familiar People | Progressive right temporal atrophy/ MRI visual assessment | 1 | Italian/ Italy | n.a. | n.a. | Person based semantic deficit, recognition deficit via tasting or smelling despite perceiving them as Italian words | Clinical observation, standardized testing for face perception, recognition, age estimation both for familiar and famous people. Formal testing for memory language executive and visuospatial functioning |
| Lambon Ralph et al., 1999^8^ \| Is a Picture Worth a Thousand Words? Evidence from Concept Definitions by Patients with Semantic Dementia | R> L temporal lobe atrophy/ Standardized visual MRI atrophy rating | 2 | n.a./ UK | n.a. | n.a. | Better performance on word-semantics than picture-semantics and producing richer definitions to words than to pictures | The PPT test |
| Perry & Hodges, 2000^9^ \| Differentiating frontal and temporal variant frontotemporal dementia from Alzheimer’s disease | tv-FTD (right predominant)/ SPECT-MRI | 2 | n.a./ UK | n.a. | n.a. | Progressive loss of memory for words and increasing difficulty recognizing familiar objects and people. Spared attention and executive function | Medical notes, formal assessment for memory, language, attention, executive and visuospatial functions |
| Lambon Ralph et al., 2001^10^ \| No right to speak? The relationship between object naming and semantic impairment: Neuropsychological evidence and a computational model | R> L temporal lobe atrophy/ Standardized visual MRI atrophy rating | 5 | n.a./ UK | n.a. | n.a. | Naming errors (mostly function description, coordinate and visual errors rather than omission) | Standardized tests for word picture matching, naming, comprehension |
| Mendez & Ghajarnia, 2001^11^ \| Agnosia for familiar faces and odors in a patient with right temporal lobe dysfunction | Right temporal atrophy/ MRI and SPECT visual assessment | 1 | n.a./ US | n.a. | n.a. | Agnosia for familiar  faces and odors | Tests for facial processing,  famous face recognition, description of person from name, and picture, the  Famous Faces Familiarity Test,  (Yes/no responses—24 alternate famous faces vs 24 unfamiliar ones)  Benton Facial Recognition (match 54 unfamiliar faces)  UPSIT odor identification, perceived intensity, pleasantness and familiarity |
| Miller et al., 2001^12^ \| Neuroanatomy of the self: Evidence from patients with frontotemporal dementia | FTD with RATL hypoperfusion / SPECT hypoperfusion | 1 | n.a./ US | n.a. | n.a. | Diminished maintenance of the self | Clinician observation |
| Mychack et al., 2001^13^ \| Novel Applications of Social-Personality Measures to the Study of Dementia | RTLV-FTD/ MRI visual scoring | 1 | n.a./ US | n.a. | n.a | Change in social personality | The Big Five Inventory, IAS and Interpersonal Measure of Psychopathy |
| Perry et al., 2001^14^ \| Hemispheric Dominance for Emotions, Empathy and Social Behaviour: Evidence from Right and Left Handers  with Frontotemporal Dementia | FTD with RTLA/ Clinical assessment, MRI atrophy rating | 2 | n.a./USA | n.a. | n.a. | Impaired recognition of  emotion from faces and voices that was not due to verbal semantic or perceptual difficulties. Loss of empathy, affected interpersonal  skills and facial expression of emotion characterized by a fixed expression, unresponsive to situations | Tests for semantic memory, coding emotional facial expression and emotional prosody |
| Rosso et al., 2001^15^ \| Complex compulsive behaviour in the temporal variant of frontotemporal dementia | FTD, temporal> Frontal, Right>Left/ MRI blind rating based on standardized scales, SPECT | 5 | n.a./ Netherlands | n.a. | n.a. | Preoccupation with certain ideas, single activities, adherence of fixed time schedule, parsimony, arranging items in a particular order, cleaning ritual | Semi structured interview |
| Simons et al., 2001^16^ \| Semantic knowledge and episodic memory for faces in semantic dementia | SD with right predominance / MRI atrophy visual scoring | 4 | n.a. | n.a. | n.a. | Face recognition memory | Recognition and memory  tests for faces |
| Gainotti et al., 2003^17^ \| Slowly progressive defect in recognition of familiar people in a patient with right anterior temporal atrophy | RATL focal neurodegeneration/ MRI atrophy assessment and SPECT | 1 | n.a./ Italy | n.a. | n.a. | Face recognition deficit | Medical notes, tests for face matching, age estimation from photographs, familiarity, and naming. Identification of friends and family members from photographs. Accessing information about celebrities through their faces and voices and names |
| Joubert et al., 2003^18^ \| Impaired configurational processing in a case of progressive prosopagnosia associated with predominant right temporal lobe atrophy | RTLA/ VBM | 1 | n.a./ France | n.a. | n.a. | Prosopagnosia, semantic memory deficit for famous monuments and famous public events (via visual stimuli, not verbal) | Tests for processing familiar and unfamiliar faces; matching, age and sex perception, facial emotion expression, learning new faces, yes/no familiarity test, naming, identification from picture and name, facial configurational processing, apparatus and stimuli and naming and identifying famous monuments from photographs and names. The famous events battery |
| Lambon Ralph et al., 2003^19^ \| Semantic dementia with category specificity: a comparative case-series study | SD, greater atrophy in the right/ MRI visual assessment | 1 | n.a./ UK | n.a. | n.a. | Semantic deficit for living things | Semantic assessment tests living vs non-living and functional vs sensory (domain, familiarity, objective age of acquisition, imageability, name agreement, phoneme length, visual complexity, (Celex frequency) as well as assessment of attribute knowledge |
| Thompson et al., 2003^20^ \| Left/right asymmetry of atrophy in semantic dementia: Behavioral–cognitive implications | R>L-SD / independent neuroradiologist blind MRI visual assessment | 11 | n.a./ UK | n.a. | n.a. | Social awkwardness, job loss, loss of insight, and difficulty with person identification as well as semantic memory deficits. | Medical notes and formal testing for memory, language, attention, executive and visuospatial functions. |
| Gorno-Tempini et al., 2004^21^ \| Cognitive and behavioral profile in a case of right anterior  temporal lobe neurodegeneration | Rtv- FTLD/ Visual assessment of the structural MRI | 1 | n.a./ USA | n.a. | n.a. | Rigid, introverted behavior, difficulty in recognizing familiar people and objects. Impairment in recognizing food by their look, flavor or name. | The NPI, IAS, NEO-FFI, IRI, Florida Affect battery. Tests for famous face confrontation naming, recognition, semantic association, famous person name to face matching. Taste (4 solutions) discrimination. Jelly Beans; spontaneous flavor naming, picture -word matching for flavors, word-picture matching for flavors. |
| Liu et al., 2004^22^ \| Behavioral disorders in the frontal and temporal variants of frontotemporal dementia | Right predominant tvFTD / Volumetric analysis | 8 | n.a./ USA | n.a. | n.a. | NPI-Sleep scores are significantly higher in tvFTD than fvFTD | The NPI |
| Snowden et al., 2004^23^ \| Knowledge of famous faces and names in semantic dementia | SD- right temporal predominant/ Visual MRI assessment | 3 | n.a./ UK | n.a. | n.a. | Face and name identification and familiarity judgement impairment | Tests for famous faces, familiarity and naming |
| Thompson et al., 2004^24^ \| Dissociating person-specific from general semantic knowledge: Roles of the left and right temporal lobes | SD -predominant right temporal / VBM | 1 | n.a./ UK | n.a. | n.a. | Deficit for person knowledge | Tests for famous people and general sematic knowledge |
| Scahill et al., 2005^25^ \| Can episodic memory tasks differentiate semantic dementia from Alzheimer's disease? | SDR / Visual MRI assessment | 9 | n.a./ UK | n.a. | n.a. | SDR group is impaired at visual (greater) and verbal memory. | The Rey figure recall, logical memory and recognition memory tests |
| Seeley et al., 2005^26^ \| The natural history of temporal variant frontotemporal dementia | RTLV-FTD / VBM z score | 6 | n.a./ USA | n.a. | n.a. | Emotional distance, irritability, and disruption of physiologic drives (sleep, appetite, libido), compulsions towards games with words and symbols, anomia for people | Clinical notes, the NPI and formal assessment for memory, executive attention, visuospatial and language functions |
| Rainville et al, 2005^27^ \| Wayfinding in familiar and unfamiliar environments in a case of progressive topographical agnosia | Progressive RTLA/ Visual MRI assessment | 1 | n.a./ Canada | n.a. | n.a. | Prosopagnosia, topographical agnosia; inability to recognize familiar and famous buildings, monuments and landmarks, mild visual agnosia for natural categories (e.g., fruits and vegetables, animals, insects, etc.) | Tests for face perception, famous faces, monuments, familiar places identification, familiarity, and naming. Spatial representation, right-left discrimination as well as the landscape test, three mountains test, wayfinding test, pointing in an architectural environment, homing vector task. |
| Garcia-Caballero et al., 2006^28^ \| Impaired facial emotion recognition in a  case of right frontotemporal dementia | FTD with right temporal predominance/ MRI visual assessment | 1 | n.a./ Spain | n.a | n.a. | Impairment  in emotion recognition causing behavioral disturbances such as approaching marginal groups. | The Modified Ekman-Friesen Emotion Recognition Test (discrimination, matching, selection and naming) |
| Joubert et al., 2006^29^ \| The right temporal lobe variant of frontotemporal dementia | rtv-FTLD / VBM | 3 | n.a./ France | n.a. | n.a. | Person based semantic deficit | The famous people test (recognize, name and provide semantic information about famous persons from their faces, their voices and their names) |
| Williams et al., 2006^30^ \| Abnormal Configural Face Perception in a Patient with Right Anterior Temporal Lobe Atrophy | RATL atrophy/ MRI visual assessment and PET hypometabolism | 1 | n.a./ Australia | n.a. | n.a. | Configural face perception deficit | Experimental configural face processing test |
| Nakachi et al., 2007^31^ \| Progressive prosopagnosia at a very early stage of frontotemporal lobar degeneration | RTLV-FTLD/ MRI visual assessment | 1 | n.a./ Japan | n.a. | n.a. | Prosopagnosia | The lexical processing test (naming and auditory comprehension for general objects), famous face recognition test, assessing information about famous people through the person’s name, retrieving the names of famous people from verbal descriptions, familiar face recognition test, identification of familiar people from voices, visual learning test. Formal testing for memory, language, attention, executive and visuospatial functions |
| Gainotti et al., 2008^32^ \| Cross-modal recognition disorders for persons and other unique entities in a patient with right fronto-temporal degeneration | Right temporal lobe degeneration/ MRI visual assessment | 1 | n.a./ Italy | n.a. | n.a. | Famous monuments and famous person recognition deficit | Tests for person and monument knowledge (perception, naming, semantic association via picture, voice and verbal definition) |
| Josephs et al., 2008^33^ \| The anatomic correlate of prosopagnosia in semantic dementia | SD- predominant right temporal atrophy/ Volumetric analysis | 15 | n.a./ USA | n.a. | 5 subjects FTLD-U | Prosopagnosia | Prosopagnosia was considered present when the clinician specifically documented that subjects had problems recognizing familiar faces, such as faces of close family members, friends, or very famous personalities (such as the current US president). Subjects who had trouble naming familiar people only without the recognition component were not considered to have prosopagnosia |
| Brambati et al., 2009^34^ \| Atrophy progression in semantic dementia with asymmetric temporal involvement: A tensor-based morphometry study | SD- RTLV/ Volumetric analysis | 13 | n.a/ USA | n.a. | n.a. | Behavioral problems | Medical records, the NPI, formal testing for memory, language, attention, executive and visuospatial functions |
| Busigny et al., 2009^35^ \| Right anterior temporal lobe atrophy and person-based semantic defect: A detailed case study | RATL atrophy/ MRI visual assessment | 1 | n.a./ Belgium | n.a. | n.a. | Person based semantic deficit | A battery for face perception and famous people identification |
| Chan et al., 2009^36^ \| The clinical profile of right temporal lobe atrophy | RTLA/ MRI visual assessment | 20 | n.a./ UK and Netherlands | n.a. | 1 case FTLD, other case AD+Lewy body disease | Episodic memory deficit, getting lost, prosopagnosia, social disinhibition, depression, altered food preference and aggressive and obsessional behavior | Medical notes, formal testing for language, memory, attention, executive and visuospatial functions and famous faces test for prosopagnosia |
| Josephs et al., 2009^37^ \| Two distinct subtypes of right temporal variant frontotemporal dementia | rtvFTD/ Atlas-based parcellation  generated temporal, frontal, and parietal grey matter volumes which were used to  identify subjects with a right temporal dominant atrophy pattern | 20 | n.a. | n.a. | Eleven of the 20 subjects  are given a pathologic or genetic diagnosis | Hyper-religiosity, stereotypy, hunger, puzzles, compulsions, indiscriminant eating, sweet tooth, topographagnosia, parkinsonism, comprehension, word finding difficulties, memory deficits, prosopagnosia, executive dysfunction, behavioral and personality changes | Medical notes |
| Zahn et al., 2009^38^ \| Social conceptual impairments in frontotemporal lobar degeneration with right anterior temporal hypometabolism | FTD with RATL hypometabolism/ FDG-PET | 10 | n.a./ US | n.a. | n.a. | Selective impairments for social concepts | The social concept discrimination task, neurobehavioral rating scale, and formal testing for language, attention, memory, executive and visuospatial functions |
| Hailstone et al., 2010^39^ \| Progressive associative phonagnosia: A neuropsychological analysis | FTD with predominant RATL atrophy/ Clinical assessment and MRI atrophy rating | 2 | n.a./ UK | n.a. | n.a. | Associative phonagnosia and prosopagnosia | Tests for famous face and voice recognition, familiarity and identification, recognition of environmental sounds and musical instruments |
| Kashibayashi et al., 2010^40^ \| Transition of Distinctive Symptoms of Semantic Dementia during Longitudinal  Clinical Observation | Right-dominant SD/ MRI visual assessment | 4 | n.a./ Japan | n.a. | n.a. | Prosopagnosia, lack of empathy,  loss of personal awareness, disinhibition, social withdrawal  of spontaneity (ceasing to pursue hobbies), stereotypic behavior  (tendency to always walk the same route or buy the same  products), mental rigidity and inflexibility | Medical notes, the Japanese standard language test of aphasia, NPI, short-memory questionnaire |
| Mendez et al., 2010^41^ \| Interhemispheric Differences in Knowledge of Animals Among Patients With Semantic Dementia | SD-Right predominant/ MRI atrophy ratings | 11 | n.a./ USA | n.a. | n.a. | Knowledge loss for living things | Tests for animal naming, animal name fluency, and semantic knowledge for animate and inanimate items |
| Mion et al., 2010^42^ \| What the left and right anterior fusiform gyri  tell us about semantic memory | SD (L<R)/ Volumetric analysis | 4 | n.a./ UK | n.a. | n.a. | Deficits in non-verbal semantics | The camel and cactus test |
| Hailstone et al., 2011^43^ \| Voice processing in dementia: a neuropsychological  and neuroanatomical analysis | SD-R sided/ MRI visual assessment | 4 | n.a./ UK | n.a. | n.a. | Voice processing deficit | Tests for voice perception (vocal size, gender, speaker discrimination) and voice recognition (familiarity, identification, naming and cross-modal matching) and equivalent measures of face and name processing |
| Hoffman & Lambon Ralph, 2011^44^ \| Reverse Concreteness Effects Are Not a Typical Feature of Semantic Dementia: Evidence for the Hub-and-Spoke Model of Conceptual Representation | SD-Right/ MRI and/or CT visual assessment | 3 | n.a./ UK | n.a. | n.a. | Comprehension deficit but better for concreate than abstract words | The Cambridge semantic and concreate-abstract test batteries (synonym judgement task, description-to-verb matching test, description-to-noun matching test, verb similarity test, Shallice & McGill Word-Picture Matching task, Mischievous Monkey Test with Pictures) |
| Coon et al., 2012^45^ \| Right temporal variant frontotemporal dementia with motor neuron disease | rtvFTD-MND/ Visual atrophy scoring; 2 independent raters | 3 | n.a./ USA | n.a. | FTLD-TDP type 3 (Mackenzie) (n=2) | Personality change with loss of empathy and insight; apathy, abnormal eating behaviors, ritualistic behaviors, becoming fearful, anomia progressing to mutism loss of semantic knowledge, topographagnosia prosopagnosia | Medical notes, formal assessment for memory, language, attention, executive, and visuospatial functions. |
| Longato et al., 2012^46^ \| Right Temporal Lobe Atrophy: A  Neuropsychological and Functional Imagery  Study | RATL atrophy/ MRI visual assessment | 14 | n.a./ France | n.a. | n.a. | Deficits in visual recognition memory, specific semantic knowledge deficit about famous people, emotion recognition problems and depression | The behavioral assessment questionnaire for behavioral problems, and formal assessment for memory, attention, executive visuospatial and language functions |
| Morais et al., 2012^47^ \| Prosopagnosia in FDT: Case report félix | FTD with RATL atrophy/ Visual atrophy assessment | 1 | n.a./ Portugal | CSF-AD ruled out | n.a. | Prosopagnosia, behavioral deficits, verbal semantics developed at later stages. | Medical notes, the Benton face perception and famous faces tests |
| Plezier et al., 2012 \| Episodic memory and the medial temporal lobe: not  all it seems. Evidence from the temporal variants of  frontotemporal dementia | FTD with right temporal atrophy/ Atrophy assessment using visual rating scales | 11 | n.a./ Netherlands | Amyloid negative | n.a. | Semantic memory deficit | Episodic memory: the visual association test. Semantic memory: the visual association naming test and animal fluency test |
| Snowden et al., 2012^48^ \| Famous people knowledge and the right and left temporal lobes | SD Right> Left/ MRI visual assessment | 4 | n.a./ UK | n.a. | n.a. | Loss of person knowledge (greater impairment for faces and visual tasks and left-sided atrophy for names and verbal tasks) | Tests for famous faces, naming, identification, familiarity, subjective familiarity, general semantics naming (locally developed picture naming), identification, identification/ semantic association and familiarity. |
| Tsuchida et al., 2012^49^ \| Neuropsychological profiles in semantic dementia patients with right-hemisphere–predominant temporal lobe atrophy | SD- right predominant temporal lobe atrophy/ MRI visual assessment | 2 | n.a./ Japan | n.a. | n.a. | Naming, word finding, comprehension of sentences and kanji writing were more spared in SD-right compared to SD-left | Medical notes, Western Aphasia Battery (WAB), Wechsler Adult Intelligence Scale-III (WAIS-III) and Wechsler Memory Scale-Revised (WMS-R) |
| Clarke et al., 2013^50^\| ‘The mind is its own place’: Amelioration of claustrophobia in a patient with semantic dementia | SD-predominantly right sided/ MRI visual assessment | 1 | n.a./ UK | n.a. | n.a. | Amelioration of claustrophobia, face recognition deficit, verbal and non-verbal semantic deficit | Clinical observation |
| Dara et al., 2013^51^ \| Impaired emotion processing from vocal and facial  cues in frontotemporal dementia compared to  right hemisphere stroke | bvFTD with predominant right temporal atrophy/ MRI visual assessment | 1 | n.a./ US | n.a. | n.a. | Selective  impairment in recognition of emotions from prosody and expression of emotions using both prosodic and  facial features | Affective prosody tests (identification, repetition, expression) from the aprosodia battery, facial expressions tests (identification, expression), empathy tests; IRI |
| Fletcher et al., 2013^52^ \| Agnosia for accents in primary progressive aphasia | SD, right predominant/ MRI visual assessment | 1 | British/ UK | n.a. | n.a. | Prosopagnosia, phonagnosia, anomia, accent agnosia | Experimental assessment for apperceptive (perception) and associative (semantic) processing of accents compared to object sounds, individual voices, and environmental sounds. Famous faces naming, bio, voices naming, voices bio, key personal attribute in visual modality. Clinical notes, standard testing on IQ, language, arithmetical, executive and spatial functioning, |
| Irish et al., 2013^53^ \| A tale of two hemispheres: contrasting socioemotional dysfunction in right- versus left-lateralised semantic dementia | Right-SD/ Review of clinical and structural neuroimaging scans by an expert team | 10 | n.a./ Australia | n.a. | n.a. | Deficits for, face identification, recognition of facial emotions and the capacity for empathic concern | The Ekman emotion recognition test, TASIT, IRI, and tests for facial matching and identification |
| Sabodash et al., 2013^54^ \| Suicidal Behavior in Dementia: A Special Risk in Semantic Dementia | SD-RATL atrophy predominant/ Standardized atrophy grading | 9 | n.a./ USA | n.a. | n.a. | Suicidal behavior | A checklist of neuropsychiatric symptoms. “Suicidal behavior” referred to more than just reports of suicidal ideation; “suicidal behavior” referred to suicide attempts or active suicidal behavior necessitating urgent psychiatric intervention. “Depression” referred to any endorsement by the patient or the caregiver of feelings or symptoms of depression. |
| Turan et al., 2013^55^ \| Aphasia, prosopagnosia and mania: a case diagnosed with right temporal variant semantic dementia | rtv-SD/ Visual assessment of MRI | 1 | n.a/ Turkey | n.a. | n.a. | Aphasia, prosopagnosia, mania | Clinical notes, the young mania rating scale, formal testing for memory, language, executive, attention and visuospatial functions |
| Wu et al., 2013^56^ \| Verbal creativity in semantic variant primary progressive aphasia | Right predominant svPPA/ VBM, 1 case is ambidexter whose hemispheric dominance is right side confirmed by MEG | 2 | n.a./ USA | n.a. | n.a. | Prosopagnosia, self-centrism, semantic deficit, increased religiosity and creativity in his poems, special interest in word jumbles, solitaire, dominos, and writing, spending 8 hours daily, rigid schedule | Clinical notes |
| Felix-Morais et al., 2014^57^ \| Frontotemporal dementia: neuroanatomical correlates of an atypical presentation | Right sided bvFTD/ MRI, SPECT and PET | 1 | n.a/ Portugal | n.a. | n.a. | Prosopagnosia | Clinical notes |
| Ge et al., 2014^58^ \| Clinical features of right temporal lobe variant of semantic dementia | Right temporal lobe variant of semantic dementia (RLTV)/ Visual atrophy rating and FDG-PET | 3 | n.a./ China | n.a. | n.a. | Prosopagnosia, getting lost, apathy, social disinhibition, stereotypy, compulsive behaviors | Clinical notes |
| Gliebus et al., 2014^59^ \| A case report of anxiety disorder preceding frontotemporal dementia with asymmetric right temporal lobe atrophy | bvFTD with right temporal atrophy/ MRI visual atrophy assessment | 1 | n.a./USA | n.a. | n.a. | Anxiety, obsessive compulsive behavior, preoccupations, getting lost | Clinical notes |
| Golden et al., 2014^60^ \| Identification of environmental sounds and melodies in syndromes of anterior temporal lobe degeneration | bvFTD RATL atrophy/ Blinded MRI visual assessment | 7 | n.a. / UK | n.a. | 4 of the cases have MAPT mutation | Nonverbal auditory semantic impairment | Experimental tests; environmental and melody sound matching |
| Grossi et al., 2014^61^ \| Structural connectivity in a single case of progressive prosopagnosia: The role of the right inferior longitudinal fasciculus | Right temporal variant FTD/ MRI visual assessment | 1 | n.a./ Italy | n.a. | n.a. | Prosopagnosia | Tests for face perception, famous face recognition, naming, semantic information, semantic information from name, false recognitions, false recognitions from name. And formal testing for memory, language, attention, executive and visuospatial functioning |
| Henry et al., 2014^62^ \| Neuropsychological, behavioral, and anatomical evolution in right temporal variant frontotemporal dementia: A longitudinal  and post-mortem single case analysis | The longitudinal results of the case published by Gorno-Tempini et al., 2004 | 1 | n.a./ USA | n.a. | TDP-C pathology | Semantic loss for foods and people and ultimately developed a pervasive semantic impairment affecting social-emotional as well as linguistic domains | Clinical notes, and formal testing for verbal semantic, memory, language, attention executive and visuospatial skills. For face flavor and behavioral assessment, see Gorno-Tempini et al., 2004 |
| Kamminga et al., 2014^63^ \| Differentiating between right-lateralised semantic  dementia and behavioural-variant frontotemporal  dementia: an examination of clinical characteristics  and emotion processing | Right-SD/ MRI assessment judged by the clinical team | 12 | n.a./ Australia | n.a. | n.a. | Prosopagnosia, obsessive and rigid personality, disinhibition, changes in diet. | Structured interview. The Ekman 60 task, face emotion matching task, emotion selection task, facial affect and identity discrimination task, face perception task, face matching test, famous faces task and formal testing for memory, language, executive, attention and visuospatial functions |
| Sabbe et al., 2014^64^ \| Obsessive compulsive behavior with right temporal variant frontotemporal dementia | rtvFTD/ MRI visual assessment | 2 | n.a./ Netherlands | n.a. | n.a. | Control compulsion and counting, clock-watching, hand-washing and frequent prayer | Medical records, formal testing for memory, language, attention, executive and visuospatial functions |
| Shany-Ur et al., 2014^65^ \| Self-awareness in neurodegenerative disease relies on neural structures mediating reward-driven attention | rtFTD/ Based on Josephs et al., 2009 | 7 | n.a./ USA | n.a. | n.a. | Impaired self-awareness, overestimating interpersonal functioning | The patient competency rating scale, rating observable functioning across 4  domains (daily living activities, cognitive, emotional control, interpersonal). |
| Smolentseva et al., 2014^66^ \| Characteristics of Cognitive and Behavioral Impairments in Patients with Semantic Dementia with Predominantly Right- and Left-Sided Cerebral Atrophy | SD-Right sided atrophy/ Visual assessment of MRI scans | 1 | n.a./ Russia | n.a. | n.a. | Prosopagnosia and behavioral problems | Clinical observations |
| Clark et al., 2015^67^ \| Temporal Variant Frontotemporal Dementia Is Associated with Globular Glial Tauopathy | rtv-SD/ Visual atrophy assessment | 1 | n.a./ UK | n.a. | FTLD- Globular Glial Tauopathy | Extrapyramidal features, disinhibition, apathy, abnormal eating behavior, episodic memory deficit | Medical notes, formal assessment for memory, language, executive attention and visuospatial functions |
| Everhart et al., 2015^68^ \| Right Temporal Lobe Atrophy: A Case That Initially Presented as Excessive Piety | FTLD-RTLV/ Visual atrophy assessment | 1 | n.a./ USA | n.a. | n.a. | Hyper-religiosity, depression, social disinhibition, prosopagnosia and emotion recognition deficit | Medical notes, face and emotion recognition tests |
| Gonzalez-Caballero et al., 2015^69^ \| Right temporal lobe variant of frontotemporal dementia | RTLV-FTD/ MRI visual assessment and SPECT | 2 | n.a./ Spain | n.a. | 1 case GRN | Olfactory, visual and auditory hallucinations, compulsive eating, tone discrimination deficit, changes in social conduct, person and emotion recognition deficit, rigid inflexible behavior | Medical notes and the short version of the Barcelona test and the frontal assessment battery |
| Kumfor et al., 2015^70^ \| Do I know you? Examining face and object memory in frontotemporal  dementia | Right lateralized SD/ Visual atrophy assessment | 3 | n.a./ Australia | n.a. | n.a. | Impaired face and object recognition | Tests for face perception, identity-matching, face recognition (The Cambridge Face Memory Task) and object recognition (The Cambridge Car Memory Task) |
| Suarez-Gonzalez & Crutch, 2015^71^ \| Relearning knowledge for people in a case of right variant frontotemporal dementia | rtvFTD / Visual atrophy assessment | 1 | Spanish/ Spain | n.a. | n.a. | Deficit in recognition of people by verbal and visual channels, and semantic loss about people | Tests for famous faces visual recognition, memory for faces (immediate and delayed recall), the Cambridge face perception and memory tests (upright and inverse), as well as semantic facts. |
| Mendez et al., 2015^72^ \| Impairments in the Face-Processing Network in Developmental Prosopagnosia and Semantic Dementia | Right temporal predominant SD/ Visual assessment of MRI scans | 5 | n.a./USA | n.a. | n.a. | Multimodal person knowledge disorder rather than visuospatial system related prosopagnosia | The Hooper visual organization and Gestalt Completion Tests, visual object and space perception battery, Benton judgement of line orientation test, Rey-Osterrirth Complex Figure Copy, visual object recognition, Benton facial recognition, Face configuration, familiarity, identification, name-to face matching, person knowledge from name, Ekman 60 Facial Emotions Tests |
| Peron et al., 2015^73^ \| Preservation of Person-Specific Semantic Knowledge in Semantic Dementia: Does Direct Personal Experience Have a Specific Role? | SD with right temporal atrophy/ Visual assessment of MRI scans | 1 | n.a./ France | n.a. | n.a. | Person specific semantic deficit | Tests for recognition (familiarity judgment) and identification (biographic information recall) of personally familiar names vs. famous names. |
| Shinagawa et al., 2015^74^ \| A Case of Musicophilia with Right Predominant Temporal Lobe Atrophy | RTLA/ Visual assessment of MRI scans | 1 | n.a./ Japan | n.a. | n.a. | Musicophilia as an instance of stereotypical behavior | Clinical observation |
| Binney et al., 2016^75^ \| Reading words and other people: A comparison of  exception word, familiar face and affect processing  in the left and right temporal variants of primary  progressive aphasia | rtvPPA/ Visual atrophy examination | 12 | n.a./USA | n.a. | n.a. | Impairment in performing  familiarity judgments on famous faces and in processing  socio-affective information such as facial expression of  emotion | The UCSF Famous Face Battery (Naming, Familiarity), CATS Face matching, CATS Affect matching, TASIT-SI-M Sincere, TASIT SI-M Sarcastic, IRI-EC, IRI-PT |
| Clark et al., 2016^76^ \| Altered Sense of Humor in Dementia | RTLA/ MRI Visual assessment | 1 | n.a./ UK | n.a. | n.a. | Altered sense of humor | The humor questionnaire |
| Gan et al., 2016^77^ \| Somatic Symptom Disorder in Semantic Dementia: The Role of Alexisomia | SD- Right predominant/ 2 raters for standardized MRI atrophy rating | 14 | n.a./ USA | n.a. | n.a. | Alexisomia | Medical notes. ‘Somatic symptoms’ were defined as the presence of one or more bodily symptoms that are distressing or result in significant disruption of daily life, and ‘somatic symptom disorder’ was defined as additionally including disproportionate and persistent thoughts, feelings, or behavior related to the somatic symptoms (at least 6 months) |
| Kumfor et al., 2016^78^ \| On the right side? A longitudinal study of  left- versus right-lateralized semantic dementia | Right-SD/ independent review of clinical and structural MRI scans by 2 expert neurologists | 9 | n.a./ Australia | n.a. | n.a. | Face and emotion recognition deficit, and abnormal behavior, motivation problems and stereotypical behavior | The ACE, CBI and tests for face and emotion processing |
| Lin et al., 2016^79^ \| Anatomical Correlates of Non-Verbal Perception in Dementia Patients | SD-R/ Clinical assessment and MRI atrophy rating | 5 | n.a./Taiwan | n.a. | n.a. | Sound perception deficits and semantic deficits for environmental sounds | Experimental tasks for pitch, melody and environmental sound perception, discrimination, matching, and naming. |
| Lo Buono et al., 2016^80^ \| Prosopagnosia as unusual presentation of semantic dementia: a case study | rtvFTLD/ Visual assessment of atrophy | 1 | n.a./ Italy | n.a. | n.a. | Cross-modality loss of person-based semantic knowledge. Deficit in familiar face recognition and a slow decline in language in the absence of behavioral alterations | Clinical observations, formal assessment for language, memory, executive attention and visuospatial functions |
| Shinagawa et al., 2016^81^ \| Neural basis of motivational approach and withdrawal behaviors in neurodegenerative disease | rtFTD/ Based on Josephs et al., 2009 | 14 | n.a./ USA | n.a. | n.a. | Abnormally low activation fun seeking scores | Behavioral inhibition/activation (BIS/BAS) scales |
| Van Mossevelde et al., 2016^82^ \| Clinical features of TBK1 carriers compared with C9orf72, GRN and non-mutation carriers in a Belgian cohort | bvFTD-right temporal/ MRI visual assessment | 3 | n.a./ Belgium | n.a. | TBK1 mutation | Disinhibition, apathy, inappropriate laughing | Clinical notes, formal testing for memory, language, attention, executive and visuospatial function and face recognition |
| Woollacott et al., 2016^83^ \| Right temporal variant frontotemporal dementia with motor neuron disease: A novel association with the C9orf72 expansion | rt-FTD/ MRI visual assessment | 1 | n.a./ UK | n.a. | C9orf72 expansion | Prosopagnosia, behavioral changes and ALS | Clinical notes, formal testing for memory, language, attention, executive and visuospatial functions and face recognition |
| Gola et al., 2017^84^ \| A neural network underlying intentional emotional facial expression in  neurodegenerative disease | rtFTD/ MRI visual assessment and based on Josephs et al., 2009 | 11 | n.a./ USA | n.a. | n.a. | Impaired intentional imitation | The intentional emotion expression task, IRI -EC, TASIT-EET, NEO-Five Factor Inventory-Extraversion subscale-Interpersonal warmth |
| Irish et al., 2017^85^ \| Damage to right medial temporal structures disrupts the capacity for scene construction—a case study | SD-Right/ Visual assessment of MRI scans | 1 | n.a./ Australia | n.a. | n.a. | Impoverished descriptions of spatially fragmented scenes. Prosopagnosia, with subjectively reported gaps in autobiographical memory and wayfinding difficulties | The scene construction task. Formal assessment for memory, language, attention, executive, visuospatial functioning, face and emotion recognition. |
| Konishi et al., 2017^86^ \| A different type of primary progressive aphasia: a case report of dysprosody and word deafness | Atypical PPA/ Clinical assessment and MRI atrophy rating, | 1 | n.a./Japan | n.a. | n.a. | Dysprosody and word deafness, song recognition deficit, | Clinical observation, speech audiometry, standard language assessment showed no abnormalities with verbal comprehension, |
| Koriath et al., 2017^87^ \| The clinical, neuroanatomical, and neuropathologic phenotype of TBK1-associated frontotemporal dementia: A longitudinal case report | rtvFTD/ VBM | 1 | n.a./ UK | n.a. | TBK-1 mutation, TDP-A pathology | Increased rigidity and obsessiveness, apathy, loss of empathy, and development of a sweet tooth | Medical notes, formal testing for memory, language, attention, executive and visuospatial functions |
| Luzzi et al., 2017^88^ \| Famous faces and voices: Differential profiles in early right and left  semantic dementia and in Alzheimer's disease | Right SD/ Clinical, structural MRI, FDG-PET assessment | 8 | Italian/ Italy | CSF amyloid | n.a. | Person specific knowledge deficit | Experimental battery for famous face and voice recognition and naming. |
| Pressman et al., 2017^89^ \| Observing conversational laughter in frontotemporal dementia | rtFTD/ Visual atrophy assessment | 14 | n.a./ USA | n.a. | n.a. | Laughing less | Recorded (video and audio) while discussing a problem in their relationship with a healthy control companion |
| Sakai et al., 2017^90^ \| Gustatory dysfunction as an early symptom of semantic dementia | Right predominant SD/ Visual atrophy assessment, SPECT | 7 | n.a./ Japan | n.a. | n.a. | Gustatory dysfunction at both the sensory and semantic levels | Gustatory threshold using Taste Disk® kit. Taste discrimination (judgment of the same or different tastes), identification test (taste-picture matching test) |
| Veronelli et al., 2017^91^ \| Geschwind Syndrome in frontotemporal lobar degeneration: Neuroanatomical and neuropsychological features over 9 years | rtvFTLD/ VBM, PET | 1 | n.a./ USA | n.a. | n.a. | Geschwind Syndrome; hyper-religiosity, hypergraphia, and poor emotional regulation (irritability, impulsivity, disinhibition, egocentric behavior) | Medical records, the NEO-FFI, GDS, Beck depression inventory-II, Beck anxiety inventory, frontal system behavioral scale, and formal testing for memory, language, attention, executive and visuospatial functions |
| Bevan-Jones et al., 2018^92^ \| [18F]AV-1451 binding in vivo mirrors the expected distribution of TDP-43 pathology in the semantic variant of primary progressive aphasia | Right SD/ Visual assessment of atrophy, PET | 2 | n.a./ UK | 1 case CSF Amyloid negative | n.a. | Rigid obsessional behaviour,  reduced empathy, prosopagnosia, anomia,  impaired single-word comprehension, surface dyslexia | Medical records, the ACE-R, FAB and PPT |
| Chen et al., 2018^93^ \| The neuropsychological profiles and semantic-critical regions of right semantic dementia | Right SD/ Volumetric laterality index | 17 | n.a./ China | n.a. | n.a. | Deficits in face recognition, social cognition, semantic, episodic and general cognitive abilities | Tests for face knowledge, reading the mind in the eyes test and formal testing for verbal semantic, language, memory, executive, attention and visuospatial functions |
| Cosseddu et al., 2018^94^ \| Multimodal Face and Voice Recognition Disorders in a Case With  Unilateral Right Anterior Temporal Lobe Atrophy | RATL atrophy/ MRI assessment | 1 | Italian/ Italy | n.a. | n.a. | Multimodal person identification deficit (through face and voice, but not through personal name), anxiety, depression, irritability, apathy, loss of empathy, lack of understanding of the other’s intentions, hyporexia (strong preference for food), ritualistic and obsessive behaviors regarding health, cleanness and tidiness | The famous people recognition  battery [identification (name, face and voice), familiarity judgement (familiarity score, semantic score, false alarms, naming)]. The NPI, Ekman emotion recognition test, and formal testing for memory, executive, language, attention visuospatial skills. |
| Koyama et al., 2018^95^ \| Caregiver Burden in Semantic Dementia with Right- and Left-Sided Predominant Cerebral Atrophy and in Behavioral-Variant Frontotemporal Dementia | SD (R>L)/ MRI visual atrophy rating | 1 | n.a./ Japan | n.a. | n.a. | Higher caregiver burden compared to left temporal counterparts | The Zarit burden interview, NPI, daily life activities questionnaire |
| Marshall et al., 2018^96^ \| Motor signatures of emotional reactivity in frontotemporal dementia | rtvFTD / Visual inspection of MRI scans | 6 | n.a./ UK | n.a. | n.a. | Globally reduced facial reactivity and also aberrant coupling of muscle reactivity to facial expression identification. | The face and gesture  recognition research network database, EMG, formal testing for memory, language, attention, executive and visuospatial functions. |
| Marshall et al., 2018^97^ \| Cardiac responses to viewing facial emotion differentiate  frontotemporal dementias | rtvFTD / visual inspection of MRI scans | 6 | n.a./UK | n.a. | n.a. | Spared cardiac reactivity | Watching dynamic, naturalistic videos of facial emotions during ECG recording. |
| Okada et al., 2018^98^ \| Early-stage right temporal lobe variant of frontotemporal dementia: 3 years of follow-up observations | RTLV-FTD/ MRI and brain perfusion single-photon emission CT (SPECT) | 1 | n.a./ Japan | n.a. | n.a. | Early symptoms; altered behavior and problems with interpersonal relationships characterized by self-centered behavior and a lack of empathy, fixation in music CDs. Developing semantic deficit overtime | Clinical observations. The mini-mental state examination, Alzheimer’s Disease assessment scale-cognitive component-Japanese version, and clock drawing test. |
| Pressman et al., 2018^99^ \| Neuroanatomy of Shared Conversational Laughter in Neurodegenerative Disease | rtvFTD/ Expert panel and structural MRI assessment | 6 | n.a. | n.a. | n.a. | Deficit in shared social laughter | An experimental task to record timing after partner’s laughter |
| Snowden et al., 2018^100^ \| Semantic dementia and the left and right temporal lobes | SD-right predominant atrophy/ MRI visual assessment | 1 | n.a./ UK | n.a. | n.a. | Poorer understanding of faces/pictures/models | Tests for famous face and name identification, animal knowledge from 3-D models and animal names, and the PPT pictures and words. |
| Woollams & Patterson, 2018^101^ \| Cognitive consequences of the left-right asymmetry of atrophy in semantic dementia | SD R>L/ Experienced neurologist clinical examination and MRI visual rating | 20 | n.a./ UK | n.a. | n.a. | Picture based semantic association deficit | Tests for picture naming (naming and errors of commission including omission, semantic, superordinate, informative circumlocution, other circumlocution, other, or unrelated), spoken word to picture matching, The PPT pictures. |
| Battista et al., 2019^102^ \| Early pathological gambling in co-occurrence with semantic variant primary progressive aphasia: a case report | svPPA (right > left)/ Visual assessment of MRI scans, DAT-SCAN, SPECT; complete lack of perfusion in the RATL | 1 | n.a./ Italy | Amyloid negative | n.a. | Early-onset pathological gambling (impulse control disorder), parsimonious | The Barratt impulsiveness scale, south oaks gambling screen, apathy scale, GDS, NPI, FBI. |
| Borghesani et al., 2019^103^ \| “Looks familiar, but I do not know who she is”: The  role of the anterior right temporal lobe in famous face recognition | Right- svPPA/ MRI visual atrophy and consensus diagnosis of the Language Neurobiology  Laboratory at UCSF based on overall clinical profile | 15 | n.a./ USA | n.a. | n.a. | Famous face processing | The UCSF famous face battery including confrontation naming, semantic association, familiarity judgement. And formal testing for memory, language, attention, executive, and visuospatial functions. |
| Chen et al., 2019^104^ \| Neural substrates of amodal and modality-specific semantic processing within the temporal lobe: A lesion-behavior mapping study of semantic dementia | Right predominant SD/ Visual assessment of MRI scans | 14 | n.a./ China | n.a. | n.a. | Non-verbal semantic processing deficit | Tests for oral picture naming, oral sound naming, picture associative matching, word associative matching, word picture verification, and formal testing for memory, language, attention, executive and visuospatial functions. |
| de Brito et al., 2019^105^ \| [18F]FDG-PET in a case of right temporal lobe variant of frontotemporal dementia | RTLV-FTD/ PET | 1 | n.a./ Brazil | AD negative | n.a. | Slowly progressive amnestic and topographical disorientation symptoms, followed by an early onset of apathy, hyperorality, ritualistic behaviors, prosopagnosia, and phonagnosia | Clinical notes |
| Kumfor et al., 2019^106^ \| Facial expressiveness and physiological arousal in frontotemporal dementia: Phenotypic clinical profiles and neural correlates | Right-SD / Visual assessment of atrophy | 5 | n.a./ Australia | n.a. | n.a. | Abnormal facial expressiveness, which was discordant with the emotional content of the stimuli | Six movies (120 sec each), with two positive (When Harry met Sally and Mr. Bean’s Christmas), two neutral (Birds and Stream documentaries), and two negative (My Bodyguard and Cry Freedom) films, during surface facial EMG and skin conductance level recording |
| Miki et al., 2019^107^ \| Corticospinal tract degeneration and temporal lobe atrophy in frontotemporal lobar degeneration TDP-43 type C pathology | Right temporal lobe dominant SD/ Visual assessment of atrophy | 1 | n.a./ UK | n.a. | TDP-C | Difficulties recognizing familiar faces, change in personality, losing interest in doing things and becoming less empathic. | Medical records, formal testing for memory, language, attention, executive, and visuospatial functions |
| Mole et al., 2019^108^ \| Avian agnosia: A window into auditory semantics | Predominantly right-sided SD / Experienced SD team clinical examination and MRI visual rating | 1 | n.a./ UK | n.a. | n.a. | Difficulties in recognition of birds by their calls rather than visually and people by their faces and voices. Accent naming problems. Memory deficits about places, lack of energy, lack of empathy, routine-bound, and inflexible restricted interests to birds and change in food preference. Slow and aprosodic speech. | Clinical observation, experimental tasks for bird ringing rating comparing with 10 local age, sex, education and experience (years) in bird ringing matched controls with expertise in bird ringing and auditory perceptional discrimination. For non-expert knowledge; picture and auditory naming, insect naming, accent naming, famous face and voice naming tests. For expert knowledge: bird picture and sound naming, bird gender discrimination and advanced bird naming tests |
| Kwon, 2019^109^ \| Temporal Variant Frontotemporal Dementia | tvFTD, right predominant/ MRI visual atrophy scoring | 1 | n.a. / Korea | n.a. | n.a. | Episodic memory deficit and disinhibition | Clinical observations and cognitive tests. Test details were not mentioned |
| Pozueta et al., 2019^110^ \| Cognitive and Behavioral Profiles of Left and Right Semantic Dementia: Differential Diagnosis with Behavioral Variant Frontotemporal Dementia and Alzheimer’s Disease \| | Right SD/ Visual rating of structural MRI. When in doubt, the FDG-PET hypometabolism pattern was used. | 18 | n.a./ Spain | Amyloid PET | n.a. | Prosopagnosia and facial emotion recognition deficit | Experimental tasks for famous face recognition, naming, semantic information about target person. Standardized tests; The NPI, Ekman emotion recognition task as well as formal testing for memory, language, attention, executive and visuospatial functions. |
| Shad et al., 2019^111^ \| Right temporal variant frontotemporal dementia misdiagnosed as schizophrenia | rtvFTD/ MRI visual assessment | 1 | White Anglo-Saxon/ USA | n.a. | n.a. | Depression, florid psychosis | Medical records |
| Snowden et al., 2019^112^ \| Naming and conceptual understanding in frontotemporal dementia | SD-right temporal atrophy/ MRI visual assessment | 8 | n.a./ UK | n.a. | n.a. | Face recognition problems in addition to language problems | The graded naming, word-picture naming tests, and classification of naming errors. |
| Bertoux et al., 2020^113^ \| When affect overlaps with concept: emotion recognition in semantic variant of primary progressive aphasia | svPPA- right sided atrophy/ MRI visual assessment | 4 | n.a./ France | n.a | n.a. | Emotion recognition deficit due to impairment of valance processing and conceptual knowledge for emotions | For facial emotion recognition, the Amsterdam dynamic facial expression set. For evaluation of emotional concepts; tasks for description, synonyms, matching, examples, and context choice |
| Borghesani et al., 2020^114^ \| Regional and hemispheric susceptibility of the temporal lobe to FTLD-TDP  type C pathology | svPPA -right predominant/ MRI visual atrophy and consensus diagnosis of the UCSF-MAC based on overall clinical profile, further studied ATL parcellation. | 12 | n.a./ USA | n.a. | All cases have FTLD TDP-43 pathology | Decreased socioemotional sensitivity, interpersonal warmth, empathy and visual semantics. | The NPI, RSMS, IAS, IRI; visual semantics PPT pictures, and formal testing for memory, language, executive, and visuospatial functions |
| Caso et al., 2020^115^ \| Temporal variant of frontotemporal dementia in C9orf72 repeat expansion carriers: two case studies | rtvFTD/ MRI visual atrophy rating | 1 | n.a./ Italy | n.a. | FTLD gene + (c9orf72), | Anomia, obsessive ritualistic behavior, lack of empathy, emotion recognition difficulties, overly strict routines and opinions, anxiety, overly friendly, word finding difficulties, landmark & famous face identification and naming deficit | Semi structures interviews and the BLED, SET, TASIT, ToM, CATS, NPI, PPT pictures and tests for face perception, famous faces naming, famous face recognition, picture naming. Additionally formal testing for memory, language, executive, visuospatial and attention functions. |
| Ding et al., 2020^116^ \| A unified neurocognitive model of semantics language social behavior and face recognition in semantic dementia | Right-SD / MRI atrophy assessment | 19 | n.a./ China | n.a. | n.a. | First with early visual face recognition or positive behavioral deficits followed by more general semantic, naming and negative behavioral problems. | The NPI and tests for oral picture naming, object naming, face naming, word picture verification, picture associative matching, object perception |
| Ulugut et al., 2020^117^ \| A clinical radiological framework of the right temporal variant of frontotemporal dementia | Right temporal variant FTD/ Structured visual MRI atrophy rating by blinded experienced neuroradiologist | 70 | n.a./ Netherlands | Amyloid positivity ruled out based on CSF and/or PET | 8 subjects had FTLD pathologies and/or genes | Prosopagnosia, memory deficit, disinhibition, apathy, loss of empathy, compulsiveness, depression, word finding and naming difficulties | Clinical notes, the NPI, GDS and formal testing for memory, language, attention, executive and visuospatial functions. |
| Wong et al., 2020^118^ \| Apathy and its impact on carer burden and psychological wellbeing in primary progressive aphasia | Right SD/ MRI atrophy assessment | 16 | n.a./ Australia | n.a. | n.a. | Apathy | The NPI, ZBI, and an apathy composite score based on the CBI-R, FrSBe scores. Formula= [CBI-R motivation % score + FrSBE apathy % score] / 2). |
| Curet Burleson et al., 2021^119^ \| Neurobehavioral Characteristics of FDG-PET Defined Right-Dominant Semantic Dementia: A Longitudinal Study | Right Dominant Semantic Dementia/ FDG-PET hypometabolism in the right temporal lobe [2 standard deviations from controls (left+ group)] | 7 | n.a./ USA | n.a. | All patients were screened for GRN, MAPT, C9orf72, results were not shared | Greater frequency of abnormal behaviors | The NPI |
| Funayama et al., 2021^120^ \| Putative Alcohol-Related Dementia as an Early Manifestation of Right Temporal Variant of Frontotemporal Dementia \| | rtvFTD/ MRI visual atrophy scoring | 1 | Japanese/ Japan | n.a. | n.a. | Alcohol dependence, excessive consumption of soft drinks, stealing food, hoarding newspapers and magazines, stereotyped fixed schedule, drawing graffiti, losing the ability to understand and share the thoughts or feelings of another, semantic memory and face recognition deficit. | ICD criteria by 2 independent psychiatrists. Similarly, he scored 0 out of 16 in the famous people naming task in the visual perception test for agnosia, Japanese version of the Wechsler Adult Intelligence Scale—Third Edition |
| Hutchings et al., 2021^121^ \| Considering Hemispheric Specialization in Emotional Face  Processing: An Eye Tracking Study in Left- and  Right-Lateralised Semantic Dementia | Right-SD/ Visual MRI assessment | 6 | n.a./ Australia | n.a. | n.a. | Increased fixation to the eyes during emotion recognition task | Facial affect selection and discrimination tests and eye tracking |
| Joo et al., 2021^122^ \| Parosmia in Right-lateralized Semantic Variant Primary Progressive Aphasia  A Case Report | Right lateralized svPPA/ Visual atrophy rating | 1 | n.a./ Korean | n.a. | n.a. | Prosopagnosia, parosmia | The Sniffin’ Stick Test, NPI, GDS and formal testing for famous faces, emotion recognition, language memory, attention, executive and visuospatial functions. |
| Sato et al., 2021^123^ \| Characteristics of behavioral symptoms in right-sided predominant semantic dementia and their impact on caregiver burden: a cross-sectional study | Right sided predominant SD/ MRI rating used in Ulugut et al., 2020 | 14 | n.a/ Japan | n.a. | n.a. | Apathy, disinhibition, stereotypical behavior and higher caregiver burden | The Japanese version of the ZBI, Lawton IADL, NPI, and Stereotypy Rating Inventory |
| Shaw et al., 2021^124^ \| Anhedonia in semantic dementia; Exploring right hemispheric contributions to the loss of pleasure | SD-Right/ MRI visual rating | 8 | n.a./ Australia | n.a. | n.a. | Anhedonia, apathy | The Snaith-Hamilton Pleasure Scale, the motivation subscale of the CBI-R, as a validated carer-rated index of apathy. |
| Ulugut et al., 2021^125^ \| The Right Temporal Variant of Frontotemporal Dementia Is Not Genetically Sporadic: A Case Series | Right temporal variant FTD/ Based on Ulugut et al., 2020 | 6 | Dutch (n=5) and Turkish (n=1)/ Netherlands | n.a. | All cases have FTLD genes | Prosopagnosia, memory deficit, disinhibition, apathy, loss of empathy, compulsiveness, depression, word finding and naming difficulties | Clinical notes, the NPI, GDS and formal testing for memory, language, attention, executive and visuospatial functions. |
| Ulugut et al., 2021^126^ \| Right temporal variant frontotemporal dementia is pathologically heterogeneous: a case-series and a systematic review | Right temporal variant FTD/ Based on Ulugut et al., 2020 | 5 | Dutch/ Netherlands | n.a. | All cases have FTLD pathologies | Prosopagnosia, memory deficit, disinhibition, apathy, loss of empathy, compulsiveness, depression, word finding and naming difficulties | Clinical notes, the NPI, GDS and formal testing for memory, language, attention, executive and visuospatial functions. |
| Carnemolla et al., 2022^127^ \| Olfactory Bulb Integrity in Frontotemporal Dementia and Alzheimer’s Disease | SD-RATL predominant/ MRI visual atrophy scoring | 10 | n.a./ Australia | n.a. | n.a. | Smell loss | Symptoms of smell loss were established from items from the CBI-R and the Appetite and Eating Habits Questionnaire. |
| Dong et al., 2022^128^ \| Genetic Spectrum and Clinical Heterogeneity of Chinese Frontotemporal Dementia Patients: Data from PUMCH Dementia Cohort | svPPA, right temporal predominant/ MRI visual assessment, PET | 2 | n.a./ China | E3E3 | TBK1, SQSTM1 | Impaired naming, comprehension and spontaneous speech, dysgraphia, depression,  stereotyped behavior, hallucination, memory deficit, insomnia, paranoid, dietary change | Medical notes, formal assessment for memory, language, attention, executive and visuospatial functions. |
| Koros et al., 2022^129^ \| Prosopagnosia, Other Specific Cognitive Deficits, and Behavioral Symptoms: Comparison between Right Temporal and Behavioral Variant of Frontotemporal Dementia | rtvFTD/ Blind visual atrophy rating | 7 | n.a./ Greece | n.a. | n.a. | Early changes; prosopagnosia, apathy, and episodic memory impairment. Later stages; social awkwardness, compulsive behaviors, disinhibition and loss of insight with a marked personality change | Medical notes and the FAB, NPI, FBI. |
| Paranhos et al., 2022^130^ \| A presumptive association  between obsessive compulsions  and asymmetric temporal lobe atrophy: a case  report | tvFTD right predominant/ Visual atrophy rating | 1 | n.a./ Brazil | n.a. | n.a. | Early symptoms, obsessive-compulsive rituals, preoccupations with cleanliness and orderliness | Medical notes, formal assessment for memory, language, attention, executive and visuospatial functions. |
| Pourriyahi et al., 2022^131^ \| “Split-day syndrome”, a patient with frontotemporal dementia who lives two days in the span of one: a case report and review of articles | FTD with right temporal atrophy/ Visual atrophy rating | 1 | n.a./ Iran | n.a. | n.a. | Anomia, self-centrism and not paying attention to others, sweet craving, hyperorality, apathy, and lack of interest in routine daily activities. Time disorientation, such as splitting each day into two, about 12-h intervals, e.g., two sets of breakfast, lunch, and dinner through every 24 h, split in half; “split-day syndrome.” | Clinical observation, standard assessment for memory, language, attention, executive and visuospatial functions. |
| Rossi et al., 2022^132^ \| Semantic and right temporal variant of FTD: Next generation sequencing genetic analysis on a single-center cohort | rtvFTD/ Based on Ulugut et al., 2020 | 8 | n.a./ Italy | CSF Amyloid status | CHCHD10 (VUS), MAPT (likely benign) PSEN1 (likely pathogenic) | Changes in personality and behavior (eating behavior changes, loss of empathy, compulsive behaviors), memory loss, prosopagnosia and topographical disorientation. | Medical notes, standard assessment for cognitive and behavioral deficits. Test details were not mentioned. |
| Younes et al., 2022^133^ \| Right temporal degeneration and socioemotional semantics: semantic behavioural variant frontotemporal dementia | sbvFTD/ W-score maps | 46 | n.a./ USA | APOE4 prevalence  E2E3=7  E3E3=22  E3E4=8 | 19 cases have FTLD pathology 2 cases have FTLD genes | Loss of empathy, person specific  semantic impairment, complex compulsions, rigid thought process, | The NPI, GDS, CATS face and affect matching, UCSF famous faces naming, familiarity, semantic association, name familiarity, TASIT-EET, TASIT SI-M sincere and sarcastic, IRI-EC, IRI-PT, emotional ToM, cognitive ToM, BIS/BAS, IAS, RSMS. Additionally, formal testing for language including verbal semantics, memory, attention, executive and visuospatial functions. |
| Di Napoli et al., 2023^134^ \| Predominant right temporal lobe atrophy: Clinical, neuropsychological and structural differences based on amyloid status | RTLA/ Structured visual MRI rating | 17 | n.a./ Italy | 7 amyloid positive, 10 negative | n.a. | Executive dysfunction and topographical disorientation were more common in amyloid-positive patients. Social awkwardness and compulsive attitude, were more frequent in the amyloid-negative patients. | Medical records, formal testing for memory, language, attention, executive and visuospatial functions |
| Frings et al., 2023^135^ \| More extensive hypometabolism and higher mortality risk in patients with right- than left-predominant neurodegeneration of the anterior temporal lobe | rtvFTD/ FDG-PET | 10 | n.a./ Germany | n.a. | n.a. | Shorter survival duration of patients with right than left ATL neurodegeneration | The German version of the Consortium to Establish a Registry for Alzheimer’s Disease Neuropsychological Assessment Battery, and mortality risk |
| Geraudie et al., 2023^136^ \| Expressive Prosody in Patients With Focal Anterior  Temporal Neurodegeneration | sbvFTD/ Based on Younes et al., 2022 | 18 | n.a./ USA | n.a. | n.a. | Decreased expressive prosodic range correlated with informant-rated empathy decline | Automatic extraction of prosody during the picture description task and the RSMS, CATS affect matching |
| Gressie et al., 2023^137^ \| Error profiles of facial emotion recognition in  frontotemporal dementia and Alzheimer’s disease | Right-SD/ Based on Kumfor et al., 2016, Ulugut et al., 2020 | 14 | n.a./ Australia | n.a. | n.a. | Emotion recognition deficit | The facial affect selection task |
| Hazelton et al., 2023^138^ \| Hemispheric contributions toward interoception and emotion recognition in left- vs right-semantic dementia \| | Right SD/ MRI atrophy rating | 6 | n.a. | n.a. | n.a. | Interoception, the perception of internal bodily cues, and emotion recognition problems | The facial affect selection task for emotion recognition. For interoception, during an ECG recording, participants were instructed to respond via button press each time they: 1) felt their own heartbeat, without physically measuring or checking their pulse (cardiac interoception); or 2) heard an audio recording of a heartbeat, based on a variable rate equivalent to an average heartbeat of 60 BPM (exteroception, control). |
| Hua et al., 2023^139^ \| Diminished baseline autonomic outflow in semantic dementia relates to  left-lateralized insula atrophy | sbvFTD/ Based on Younes et al., 2022 | 13 | European American=11, Asian American=1, Hispanic American=1 / USA | n.a. | n.a. | Impaired baseline autonomic nervous system physiology | Measurement for respiratory sinus arrhythmia (parasympathetic measure) and skin conductance level (sympathetic measure) and the IAS |
| Kawakatsu et al., 2023^140^ \| Clinicopathological diversity of semantic dementia: Comparisons of patients with early-onset versus late-onset, left-sided versus right-sided temporal atrophy, and TDP-type A versus type C pathology | Right predominant SD/ Volumetric analysis of MRI and SPECT | 2 | n.a./ Japan | Case 1: n.a.  Case 2: E3/E3 | Case 1: TDP-C  Case 2: TDP-A and LBD | Impaired understanding of objects and faces, suicidal thoughts, depression, obsessive pessimistic preoccupations. Shopping at the same time every day and buying many of the same items. Bathing 10 times per day. Wearing bizarre dark makeup, bradykinesia, visual hallucinations | Tests for famous faces as well as standard assessment for memory, language, attention, executive and visuospatial functions. |
| Leocadi et al., 2023^141^ \| Brain structural abnormalities and cognitive changes in a patient with 17q21.31 microduplication and early onset dementia: a case report | Early onset dementia with right temporal atrophy/ Volumetric assessment | 1 | Italian/ Italy | Increased tau and phospo-tau, normal amyloid | 17q21.31 microduplication | Anosognosia, impulsivity, apathy and aggressiveness | Medical notes, the SET, and formal testing for memory, language, attention, executive and visuospatial functioning |
| Mesulam et al., 2023^142^ \| Frontotemporal Degeneration with Transactive Response DNA-Binding Protein Type C at the Anterior Temporal Lobe | Right PPA or Right ATL bvFTD/ Visual and volumetric assessment of MRI | 10 | n.a./ USA | n.a. | All cases have FTLD TDP-C pathology | Impairment in social conduct, naming, object recognition, person identification, and word comprehension. Profound loss of empathy, rigidity of comportment, and bizarre food preferences. Co-occurrence of additional naming, word comprehension, and face recognition impairments. | Medical records and standard assessment for memory, language, executive, visuospatial and attention functions |
| Ohm et al., 2023^143^ \| Neuroanatomical and cellular degeneration associated with a social disorder characterized by new ritualistic belief systems in a TDP-C patient vs. a Pick patient | Right predominant temporal atrophy/ MRI visual assessment | 1 | White/ USA | n.a. | FTLD-tau | Compulsive, goal-directed behaviors related to general themes of positivity and spirituality | Clinical notes |
| Piccininni et al., 2023^144^ \| Which components of famous people recognition are lateralized? A study of  face, voice and name recognition disorders in patients with neoplastic or  degenerative damage of the right or left anterior temporal lobes | RATL neurodegeneration/ Clinical and neuroimaging (MRI, CT, PET) assessment | 8 | n.a. /Italy | n.a. | n.a. | Famous people recognition deficit | The famous people recognition battery (face, voice, name); recognition, familiarity, semantic association, naming. |
| Ramanan et al., 2023^145^ \| Mapping behavioural, cognitive and affective  transdiagnostic dimensions  in frontotemporal dementia | SD-right/ Magnitude and laterality of ATL and temporopolar atrophy on structural MRI | 11 | n.a./ Australia | n.a. | n.a. | Semantic dysfunction, visuospatial changes | The CBI-R, NPI, ZBI, emotion recognition and affect selection test, and formal assessment for memory, language, attention, executive and visuospatial functions |
| Ghirelli et al., 2024^146^ \| Clinical and neuroanatomical characterization of the semantic behavioral variant of frontotemporal dementia in a multicenter Italian cohort | sbvFTD / Based on Younes et al., 2022 | 15 | n.a./ Italian | n.a. | C9orf72 (N = 1), GRN (N = 1), MAPT (N = 1) | Early symptoms: Word, object and person-specific semantic loss, complex compulsions and rigid thought. Later symptoms: apathy/ inertia, loss of empathy, anxiety, suspiciousness | Medical notes, the BLED, cognitive estimation task, Benton face recognition test, famous face naming test |

Comparative Large Cohort Studies. Small-Group Analyses or Case Series. Individual Case Reports

R: right, L: left, rt: right temporal, rtv: right temporal variant, RTLV: right temporal lobe variant, RATL: right anterior temporal lobe, RTLA: right temporal lobe atrophy, tv: temporal variant, fv: frontal variant, FTD: frontotemporal dementia, FTLD: frontotemporal lobar degeneration, SD: semantic dementia, PPA: primary progressive aphasia, svPPA: semantic variant primary progressive aphasia, bvFTD: behavioral variant frontotemporal dementia, sbvFTD: semantic behavioral variant frontotemporal dementia, AD: Alzheimer’s Disease, MRI: magnetic resonance imaging, PET: positron emission tomography, SPECT; single photon emission computed tomography, EMG: electromyography, ECG: electrocardiogram, RSMS: Revised Self-monitoring Scale, IRI: Interpersonal Reactivity Index, EC: Empathic concern, PT: Perspective taking, IAS: Interpersonal Adjective Scales, BIS/BAS: Behavioural Inhibition System/Behavioural Activation System, TASIT: The Awareness of Social Inference Test, EET: Emotion Evaluation Test, SI-M: Social Inference–Minimal Test, CATS: Comprehensive Affect Testing System, NEO-FFI: NEO Five-Factor Inventory, NPI: Neuropsychiatric Inventory, FAB: Frontal Assessment Battery, PPT: Pyramids and Palm Trees Test, ACE-R: Addenbrooke’s Cognitive Examination-Revised, ACE-III: Addenbrooke’s Cognitive Examination- Third edition, UPSIT: University of Pennsylvania Smell Identification Test, BLED: Battery for the Assessment of Language in the Right Hemisphere, SET: Story-based Empathy Test, ToM: Theory of Mind, CBI-R: Cambridge Behavioral Inventory-Revised, FrSBe: Frontal Systems Behavioral Scale, ZBI: Zarit Burden Interview, IADL: Instrument Activities of Daily Living, GDS: Geriatric Depression Scale, FBI: Frontal Behavioral Inventory, VUS: Variants of unknown significance

REFERENCES

1 Tyrrell PJ, Warrington EK, Frackowiak RSJ, Rossor MN. Progressive degeneration of the right temporal lobe studied with positron emission tomography. *J Neurol Neurosurg Psychiatry* 1990; **53**: 1046–50.

2 Barbarotto R, Capitani E, Spinnler H, Trivelli C. Slowly progressive semantic impairment with category specificity. *Neurocase* 1995; **1**: 107–19.

3 Evans JJ, Heggs AJ, Antoun N, Hodges JR. Progressive prosopagnosia associated with selective right temporal lobe atrophy. A new syndrome? *Brain J Neurol* 1995; **118 ( Pt 1)**: 1–13.

4 Kazui H, Tanabe H, Ikeda M, Hashimoto M, Yamada N. A case of predominantly right-temporal lobe atrophy with disturbance of identifying familiar faces. *Brain Nerve* 1995; **47**: 77–85.

5 Edwards-Lee T, Miller BL, Benson DF, *et al.* The temporal variant of frontotemporal dementia. *Brain J Neurol* 1997; **120 ( Pt 6)**: 1027–40.

6 Miller BL, Cummings J, Mishkin F, *et al.* Emergence of artistic talent in frontotemporal dementia. *Neurology* 1998; **51**: 978–82.

7 Gentileschi V, Sperber S, Spinnler H. Progressive defective recognition of familiar people. *Neurocase* 1999; **5**: 407–24.

8 Lambon Ralph MA, Graham KS, Patterson K, Hodges JR. Is a Picture Worth a Thousand Words? Evidence from Concept Definitions by Patients with Semantic Dementia. *Brain Lang* 1999; **70**: 309–35.

9 Perry RJ, Hodges JR. Differentiating frontal and temporal variant frontotemporal dementia from Alzheimer’s disease. *Neurology* 2000; **54**: 2277–84.

10 Ralph MAL, McClelland JL, Patterson K, Galton CJ, Hodges JR. No Right to Speak? The Relationship between Object Naming and Semantic Impairment:Neuropsychological Evidence and a Computational Model. *J Cogn Neurosci* 2001; **13**: 341–56.

11 Mendez MF, Ghajarnia M. Agnosia for familiar faces and odors in a patient with right temporal lobe dysfunction. *Neurology* 2001; **57**: 519–21.

12 Miller BL, Seeley WW, Mychack P, Rosen HJ, Mena I, Boone K. Neuroanatomy of the self. *Neurology* 2001; **57**: 817–21.

13 Mychack P, Rosen H, Miller BL. Novel applications of social-personality measures to the study of dementia. *Neurocase* 2001; **7**: 131–43.

14 Perry RJ, Rosen HR, Kramer JH, Beer JS, Levenson RL, Miller BL. Hemispheric Dominance for Emotions, Empathy and Social Behaviour: Evidence from Right and Left Handers with Frontotemporal Dementia. *Neurocase* 2001; **7**: 145–60.

15 Rosso SM, Roks G, Stevens M, *et al.* Complex compulsive behaviour in the temporal variant of frontotemporal dementia. *J Neurol* 2001; **248**: 965–70.

16 Simons JS, Graham KS, Galton CJ, Patterson K, Hodges JR. Semantic knowledge and episodic memory for faces in semantic dementia. *Neuropsychology* 2001; **15**: 101–14.

17 Gainotti G, Barbier A, Marra C. Slowly progressive defect in recognition of familiar people in a patient with right anterior temporal atrophy. *Brain J Neurol* 2003; **126**: 792–803.

18 Joubert S, Felician O, Barbeau E, *et al.* Impaired configurational processing in a case of progressive prosopagnosia associated with predominant right temporal lobe atrophy. *Brain* 2003; **126**: 2537–50.

19 Lambon Ralph MA, Patterson K, Garrard P, Hodges JR. Semantic Dementia with Category Specificity:acomparative Case-Series Study. *Cogn Neuropsychol* 2003; **20**: 307–26.

20 Thompson SA, Patterson K, Hodges JR. Left/right asymmetry of atrophy in semantic dementia: behavioral-cognitive implications. *Neurology* 2003; **61**: 1196–203.

21 Gorno-Tempini ML, Rankin KP, Woolley JD, Rosen HJ, Phengrasamy L, Miller BL. Cognitive and Behavioral Profile in a Case of Right Anterior Temporal Lobe Neurodegeneration. *Cortex* 2004; **40**: 631–44.

22 Liu W, Miller BL, Kramer JH, *et al.* Behavioral disorders in the frontal and temporal variants of frontotemporal dementia. *Neurology* 2004; **62**: 742–8.

23 Snowden JS, Thompson JC, Neary D. Knowledge of famous faces and names in semantic dementia. *Brain J Neurol* 2004; **127**: 860–72.

24 Thompson SA, Graham KS, Williams G, Patterson K, Kapur N, Hodges JR. Dissociating person-specific from general semantic knowledge: Roles of the left and right temporal lobes. *Neuropsychologia* 2004; **42**: 359–70.

25 Scahill VL, Hodges JR, Graham KS. Can episodic memory tasks differentiate semantic dementia from Alzheimer’s disease? *Neurocase* 2005; **11**: 441–51.

26 Seeley WW, Bauer AM, Miller BL, *et al.* The natural history of temporal variant frontotemporal dementia. *Neurology* 2005; **64**: 1384–90.

27 Rainville C, Joubert S, Felician O, Chabanne V, Ceccaldi M, Péruch P. Wayfinding in familiar and unfamiliar environments in a case of progressive topographical agnosia. *Neurocase* 2005; **11**: 297–309.

28 García-Caballero A, González-Hermida J, García-Lado I, Recimil MJ. Impaired facial emotion recognition in a case of right frontotemporal dementia. *Actas Esp Psiquiatr* 2006; **34**: 416–9.

29 Joubert S, Felician O, Barbeau E, *et al.* The right temporal lobe variant of frontotemporal dementia. *J Neurol* 2006; **253**: 1447–58.

30 Williams MA, Savage G, Halmagyl M. Abnormal configural face perception in a patient with right anterior temporal lobe atrophy. *Neurocase* 2006; **12**: 286–91.

31 Nakachi R, Muramatsu T, Kato M, *et al.* Progressive prosopagnosia at a very early stage of frontotemporal lobar degeneration. *Psychogeriatrics* 2007; **7**: 155–62.

32 Gainotti G, Ferraccioli M, Quaranta D, Marra C. Cross-modal recognition disorders for persons and other unique entities in a patient with right fronto-temporal degeneration. *Cortex* 2008; **44**: 238–48.

33 Josephs KA, Whitwell JL, Vemuri P, *et al.* The anatomic correlate of prosopagnosia in semantic dementia. *Neurology* 2008; **71**: 1628–33.

34 Brambati SM, Rankin KP, Narvid J, *et al.* Atrophy progression in semantic dementia with asymmetric temporal involvement: A tensor-based morphometry study. *Neurobiol Aging* 2009; **30**: 103–11.

35 Busigny T, Robaye L, Dricot L, Rossion B. Right anterior temporal lobe atrophy and person-based semantic defect: A detailed case study. *Neurocase* 2009; **15**: 485–508.

36 Chan D, Anderson V, Pijnenburg Y, *et al.* The clinical profile of right temporal lobe atrophy. *Brain J Neurol* 2009; **132**: 1287–98.

37 Josephs KA, Whitwell JL, Knopman DS, *et al.* Two distinct subtypes of right temporal variant frontotemporal dementia. *Neurology* 2009; **73**: 1443–50.

38 Zahn R, Moll J, Iyengar V, *et al.* Social conceptual impairments in frontotemporal lobar degeneration with right anterior temporal hypometabolism. *Brain* 2009; **132**: 604–16.

39 Hailstone JC, Crutch SJ, Vestergaard MD, Patterson RD, Warren JD. Progressive associative phonagnosia: A neuropsychological analysis. *Neuropsychologia* 2010; **48**: 1104–14.

40 Kashibayashi T, Ikeda M, Komori K, *et al.* Transition of distinctive symptoms of semantic dementia during longitudinal clinical observation. *Neurosci Res* 2010; **68**: e191.

41 Mendez MF, Kremen SA, Tsai P-H, Shapira JS. Interhemispheric differences in knowledge of animals among patients with semantic dementia. *Cogn Behav Neurol* 2010; **23**: 240–6.

42 Mion M, Patterson K, Acosta-Cabronero J, *et al.* What the left and right anterior fusiform gyri tell us about semantic memory. *Brain J Neurol* 2010; **133**: 3256–68.

43 Hailstone JC, Ridgway GR, Bartlett JW, *et al.* Voice processing in dementia: A neuropsychological and neuroanatomical analysis. *Brain* 2011; **134**: 2535–47.

44 Hoffman P, Lambon Ralph MA. Reverse Concreteness Effects Are Not a Typical Feature of Semantic Dementia: Evidence for the Hub-and-Spoke Model of Conceptual Representation. *Cereb Cortex* 2011; **21**: 2103–12.

45 Coon EA, Whitwell JL, Parisi JE, Dickson DW, Josephs KA. Right temporal variant frontotemporal dementia with motor neuron disease. *J Clin Neurosci* 2012; **19**: 85–91.

46 Longato N, Jung B, Noblet V, *et al.* Right Temporal Lobe Atrophy: A Neuropsychological and Functional Imagery Study (P03.083). *Neurology* 2012; **78**: P03.083-P03.083.

47 Morais R, Duro D, Parra J, *et al.* Prosopagnosia in FDT: Case report félix. *Dement Geriatr Cogn Disord* 2012; **34**: 162–3.

48 Snowden JS, Thompson JC, Neary D. Famous people knowledge and the right and left temporal lobes. *Behav Neurol* 2012; **25**: 35–44.

49 Tsuchida T, Hayashi T, Saito N, *et al.* P1-176: Neuropsychological profiles in semantic dementia patients with right-hemisphere–predominant temporal lobe atrophy. *Alzheimers Dement* 2012; **8**: P169–P169.

50 Clarke C, Fletcher P, Cifelli A, Warren JD. ‘The mind is its own place’: Amelioration of claustrophobia in a patient with semantic dementia. *J Neurol Neurosurg Psychiatry* 2013; **84**. DOI:10.1136/jnnp-2013-306573.82.

51 Dara C, Kirsch-Darrow L, Ochfeld E, *et al.* Impaired emotion processing from vocal and facial cues in frontotemporal dementia compared to right hemisphere stroke. *Neurocase* 2013; **19**: 10.1080/13554794.2012.701641.

52 Fletcher PD, Downey LE, Agustus JL, *et al.* Agnosia for accents in primary progressive aphasia. *Neuropsychologia* 2013; **51**: 1709–15.

53 Irish M, Kumfor F, Hodges JR, Piguet O. A tale of two hemispheres: contrasting socioemotional dysfunction in right- versus left-lateralised semantic dementia. *Dement Neuropsychol* 2013; **7**: 88–95.

54 Sabodash V, Mendez MF, Fong S, Hsiao JJ. Suicidal behavior in dementia: a special risk in semantic dementia. *Am J Alzheimers Dis Other Demen* 2013; **28**: 592–9.

55 Turan Ç, Kesebir S, Meteris H, Ülker M. Aphasia, prosopagnosia and mania: a case diagnosed with right temporal variant semantic dementia. *Türk Psikiyatri Derg Turk J Psychiatry* 2013; **24**: 68–72.

56 Wu TQ, Miller ZA, Adhimoolam B, *et al.* Verbal creativity in semantic variant primary progressive aphasia. *Neurocase* 2015; **21**: 73–8.

57 Felix-Morais R, Letra L, Duro D, Santana I. Frontotemporal dementia: Neuroanatomical correlates of an atypical presentation. *BMJ Case Rep* 2014. DOI:10.1136/bcr-2014-205089.

58 Ge F, Zhang Z, Li Y, *et al.* Clinical features of right temporal lobe variant of semantic dementia. *Chin J Neurol* 2014; **47**: 293–8.

59 Gliebus G. A case report of anxiety disorder preceding frontotemporal dementia with asymmetric right temporal lobe atrophy. *SAGE Open Med Case Rep* 2014; **2**: 2050313X13519977.

60 Golden HL, Downey LE, Fletcher PD, *et al.* Identification of environmental sounds and melodies in syndromes of anterior temporal lobe degeneration. *J Neurol Sci* 2015; **352**: 94–8.

61 Grossi D, Soricelli A, Ponari M, *et al.* Structural connectivity in a single case of progressive prosopagnosia: The role of the right inferior longitudinal fasciculus. *Cortex* 2014; **56**: 111–20.

62 Henry ML, Wilson SM, Ogar JM, *et al.* Neuropsychological, behavioral, and anatomical evolution in right temporal variant frontotemporal dementia: A longitudinal and post-mortem single case analysis. *Neurocase* 2014; **20**: 10.1080/13554794.2012.732089.

63 Kamminga J, Kumfor F, Burrell JR, Piguet O, Hodges JR, Irish M. Differentiating between right-lateralised semantic dementia and behavioural-variant frontotemporal dementia: an examination of clinical characteristics and emotion processing. *J Neurol Neurosurg Psychiatry* 2015; **86**: 1082–8.

64 Sabbe T, Vandenbulcke M. Obsessief-compulsief gedrag bij de rechter temporale variant van frontotemporale dementie. *Tijdschr Voor Psychiatr* 2014; : 685–8.

65 Shany-Ur T, Lin N, Rosen HJ, Sollberger M, Miller BL, Rankin KP. Self-awareness in neurodegenerative disease relies on neural structures mediating reward-driven attention. *Brain J Neurol* 2014; **137**: 2368–81.

66 Smolentseva IG, Sozinova EV, Vasenina EE, Levin OS. Characteristics of cognitive and behavioral impairments in patients with semantic dementia with predominantly right- and left-sided cerebral atrophy. *Neurosci Behav Physiol* 2014; **44**: 69–75.

67 Clark CN, Lashley T, Mahoney CJ, Warren JD, Revesz T, Rohrer JD. Temporal Variant Frontotemporal Dementia Is Associated with Globular Glial Tauopathy. *Cogn Behav Neurol* 2015; **28**: 92–7.

68 Everhart DE, Watson EM, Bickel KL, Stephenson AJ. Right Temporal Lobe Atrophy: A Case That Initially Presented as Excessive Piety. *Clin Neuropsychol* 2015; **29**: 1053–67.

69 González-Caballero G, Abellán-Miralles I, Sáenz-Sanjuan MJ. Right temporal lobe variant of frontotemporal dementia. *J Clin Neurosci* 2015; **22**: 1139–43.

70 Kumfor F, Hutchings R, Irish M, *et al.* Do I know you? Examining face and object memory in frontotemporal dementia. *Neuropsychologia* 2015; **71**: 101–11.

71 Suárez-González A, Crutch SJ. Relearning knowledge for people in a case of right variant frontotemporal dementia. *Neurocase* 2016; **22**: 130–4.

72 Mendez MF, Ringman JM, Shapira JS. Impairments in the Face-Processing Network in Developmental Prosopagnosia and Semantic Dementia. *Cogn Behav Neurol* 2015; **28**: 188–97.

73 Péron JA, Piolino P, Le Moal-Boursiquot S, *et al.* Preservation of person-specific semantic knowledge in semantic dementia: Does direct personal experience have a specific role? *Front Hum Neurosci* 2015; **9**. DOI:10.3389/fnhum.2015.00625.

74 Shinagawa S, Nakayama K. [A Case of Musicophilia with Right Predominant Temporal Lobe Atrophy]. *Brain Nerve Shinkei Kenkyu No Shinpo* 2015; **67**: 1443–8.

75 Binney RJ, Henry ML, Babiak M, *et al.* Reading words and other people: A comparison of exception word, familiar face and affect processing in the left and right temporal variants of primary progressive aphasia. *Cortex J Devoted Study Nerv Syst Behav* 2016; **82**: 147–63.

76 Clark CN, Nicholas JM, Gordon E, *et al.* Altered Sense of Humor in Dementia. *J Alzheimers Dis*; **49**: 111–9.

77 Gan JJ, Lin A, Samimi MS, Mendez MF. Somatic Symptom Disorder in Semantic Dementia: The Role of Alexisomia. *Psychosomatics* 2016; **57**: 598–604.

78 Kumfor F, Landin-Romero R, Devenney E, *et al.* On the right side? A longitudinal study of left- versus right-lateralized semantic dementia. *Brain J Neurol* 2016; **139**: 986–98.

79 Lin P-H, Chen H-H, Chen N-C, *et al.* Anatomical Correlates of Non-Verbal Perception in Dementia Patients. *Front Aging Neurosci* 2016; **8**: 207.

80 Lo Buono V, Corallo F, Marra A, Calabrò RS, Bramanti P, Marino S. Prosopagnosia as unusual presentation of semantic dementia: a case study. *Acta Clin Belg Int J Clin Lab Med* 2016; **71**: 356–8.

81 Frontotemporal dementias: From molecular mechanisms to therapy: Journal of Neurochemistry: Vol 138, No S1. https://onlinelibrary.wiley.com/toc/14714159/138/S1 (accessed May 31, 2024).

82 Van Mossevelde S, van der Zee J, Gijselinck I, *et al.* Clinical features of TBK1 carriers compared with C9orf72, GRN and non-mutation carriers in a Belgian cohort. *Brain* 2016; **139**: 452–67.

83 Woollacott I, Dick K, Gordon E, *et al.* Right temporal variant frontotemporal dementia with motor neuron disease: A novel association with the C9orf72 expansion. *J Neurochem* 2016; **138**: 311.

84 Gola KA, Shany-Ur T, Pressman P, *et al.* A neural network underlying intentional emotional facial expression in neurodegenerative disease. *NeuroImage Clin* 2017; **14**: 672–8.

85 Irish M, Mothakunnel A, Dermody N, Wilson N-A, Hodges JR, Piguet O. Damage to right medial temporal structures disrupts the capacity for scene construction—a case study. *Hippocampus* 2017; **27**: 635–41.

86 19th IPA International Congress, 31 August - 3 September 2019 Santiago de Compostela, Spain. *Int Psychogeriatr* 2019; **31**: i–172.

87 Koriath CAM, Bocchetta M, Brotherhood E, *et al.* The clinical, neuroanatomical, and neuropathologic phenotype of TBK1-associated frontotemporal dementia: A longitudinal case report. *Alzheimers Dement Diagn Assess Dis Monit* 2017; **6**: 75–81.

88 Luzzi S, Baldinelli S, Ranaldi V, *et al.* Famous faces and voices: Differential profiles in early right and left semantic dementia and in Alzheimer’s disease. *Neuropsychologia* 2017; **94**: 118–28.

89 Pressman PS, Simpson M, Gola K, *et al.* Observing conversational laughter in frontotemporal dementia. *J Neurol Neurosurg Psychiatry* 2017; **88**: 418–24.

90 Sakai M, Kazui H, Shigenobu K, Komori K, Ikeda M, Nishikawa T. Gustatory Dysfunction as an Early Symptom of Semantic Dementia. *Dement Geriatr Cogn Disord EXTRA* 2017; **7**: 395–405.

91 Veronelli L, Makaretz SJ, Quimby M, Dickerson BC, Collins JA. Geschwind Syndrome in frontotemporal lobar degeneration: Neuroanatomical and neuropsychological features over 9 years. *Cortex J Devoted Study Nerv Syst Behav* 2017; **94**: 27–38.

92 Bevan-Jones WR, Cope TE, Jones PS, *et al.* [18F]AV-1451 binding in vivo mirrors the expected distribution of TDP-43 pathology in the semantic variant of primary progressive aphasia. *J Neurol Neurosurg Psychiatry* 2018; **89**: 1032–7.

93 Chen K, Ding J, Lin B, *et al.* The neuropsychological profiles and semantic-critical regions of right semantic dementia. *NeuroImage Clin* 2018; **19**: 767–74.

94 Cosseddu M, Gazzina S, Borroni B, Padovani A, Gainotti G. Multimodal face and voice recognition disorders in a case with unilateral right anterior temporal lobe atrophy. *Neuropsychology* 2018; **32**: 920–30.

95 Koyama A, Hashimoto M, Fukuhara R, *et al.* Caregiver Burden in Semantic Dementia with Right- and Left-Sided Predominant Cerebral Atrophy and in Behavioral-Variant Frontotemporal Dementia. *Dement Geriatr Cogn Disord Extra* 2018; **8**: 128–37.

96 Marshall CR, Hardy CJD, Russell LL, *et al.* Motor signatures of emotional reactivity in frontotemporal dementia. *Sci Rep* 2018; **8**: 1030.

97 Marshall CR, Hardy CJD, Allen M, *et al.* Cardiac responses to viewing facial emotion differentiate frontotemporal dementias. *Ann Clin Transl Neurol* 2018; **5**: 687–96.

98 Okada A, Ohyama K, Ueda T. Early-stage right temporal lobe variant of frontotemporal dementia: 3 years of follow-up observations. *Case Rep* 2018; **2018**: bcr.

99 Pressman PS, Shdo S, Simpson M, *et al.* Neuroanatomy of Shared Conversational Laughter in Neurodegenerative Disease. *Front Neurol* 2018; **9**: 464.

100 Snowden JS, Harris JM, Thompson JC, *et al.* Semantic dementia and the left and right temporal lobes. *Cortex J Devoted Study Nerv Syst Behav* 2018; **107**: 188–203.

101 Woollams AM, Patterson K. Cognitive consequences of the left-right asymmetry of atrophy in semantic dementia. *Cortex J Devoted Study Nerv Syst Behav* 2018; **107**: 64–77.

102 Battista P, Capozzo R, Rizzo G, *et al.* Early pathological gambling in co-occurrence with semantic variant primary progressive aphasia: a case report. *Clin Interv Aging* 2019; **14**: 727–33.

103 Borghesani V, Narvid J, Battistella G, *et al.* ‘Looks familiar, but I do not know who she is’: The role of the anterior right temporal lobe in famous face recognition. *Cortex J Devoted Study Nerv Syst Behav* 2019; **115**: 72–85.

104 Chen Y, Chen K, Ding J, *et al.* Neural substrates of amodal and modality-specific semantic processing within the temporal lobe: A lesion-behavior mapping study of semantic dementia. *Cortex* 2019; **120**: 78–91.

105 de Brito MH, Teixeira TBM, Zampieri PF, Coutinho AM, Brucki SMD. [18F]FDG-PET in a case of right temporal lobe variant of frontotemporal dementia. *Dement Neuropsychol* 2019; **13**: 350–1.

106 Kumfor F, Hazelton JL, Rushby JA, Hodges JR, Piguet O. Facial expressiveness and physiological arousal in frontotemporal dementia: Phenotypic clinical profiles and neural correlates. *Cogn Affect Behav Neurosci* 2019; **19**: 197–210.

107 Miki Y, Ling H, Crampsie S, *et al.* Corticospinal tract degeneration and temporal lobe atrophy in frontotemporal lobar degeneration TDP-43 type C pathology. *Neuropathol Appl Neurobiol* 2020; **46**: 296–9.

108 Mole JA, Baker IW, Ottley Munoz JM, Danby M, Warren JD, Butler CR. Avian agnosia: A window into auditory semantics. *Neuropsychologia* 2019; **134**: 107219.

109 Kwon K-Y. Temporal Variant Frontotemporal Dementia. *Neurol India* 2019; **67**: 1395.

110 Pozueta A, Lage C, García-Martínez M, *et al.* Cognitive and Behavioral Profiles of Left and Right Semantic Dementia: Differential Diagnosis with Behavioral Variant Frontotemporal Dementia and Alzheimer’s Disease. *J Alzheimers Dis* 2019; **72**: 1129–44.

111 Shad MU, Howard L, Thomas K, Aga VM. Right temporal variant frontotemporal dementia misdiagnosed as schizophrenia. *Curr Psychiatry Res Rev* 2019; **15**: 223–7.

112 Snowden JS, Harris JM, Saxon JA, *et al.* Naming and conceptual understanding in frontotemporal dementia. *Cortex* 2019; **120**: 22–35.

113 Bertoux M, Duclos H, Caillaud M, *et al.* When affect overlaps with concept: emotion recognition in semantic variant of primary progressive aphasia. *Brain* 2020; **143**: 3850–64.

114 Borghesani V, Battistella G, Mandelli ML, *et al.* Regional and hemispheric susceptibility of the temporal lobe to FTLD-TDP type C pathology. *NeuroImage Clin* 2020; **28**: 102369.

115 Caso F, Agosta F, Magnani G, *et al.* Temporal variant of frontotemporal dementia in C9orf72 repeat expansion carriers: two case studies. *Brain Imaging Behav* 2020; **14**: 336–45.

116 Ding J, Chen K, Liu H, *et al.* A unified neurocognitive model of semantics language social behaviour and face recognition in semantic dementia. *Nat Commun* 2020; **11**. DOI:10.1038/s41467-020-16089-9.

117 Ulugut Erkoyun H, Groot C, Heilbron R, *et al.* A clinical-radiological framework of the right temporal variant of frontotemporal dementia. *Brain J Neurol* 2020; **143**: 2831–43.

118 Wong S, Irish M, Husain M, Hodges JR, Piguet O, Kumfor F. Apathy and its impact on carer burden and psychological wellbeing in primary progressive aphasia. *J Neurol Sci* 2020; **416**: 117007.

119 Curet Burleson AX, Pham NTT, Buciuc M, *et al.* Neurobehavioral Characteristics of FDG-PET Defined Right-Dominant Semantic Dementia: A Longitudinal Study. *Dement Geriatr Cogn Disord* 2021; **50**: 17–28.

120 Funayama M, Nakajima A, Kurose S, Takata T. Putative Alcohol-Related Dementia as an Early Manifestation of Right Temporal Variant of Frontotemporal Dementia. *J Alzheimers Dis* 2021; **83**: 531–7.

121 Hutchings R, Palermo R, Hazelton JL, Piguet O, Kumfor F. Considering hemispheric specialization in emotional face processing: An eye tracking study in left-and right-lateralised semantic dementia. *Brain Sci* 2021; **11**. DOI:10.3390/brainsci11091195.

122 Joo JY, Kim H-G, Lee KM, *et al.* Parosmia in right-lateralized semantic variant primary progressive aphasia a case report. *Alzheimer Dis Assoc Disord* 2021; **35**: 160–3.

123 Sato S, Hashimoto M, Yoshiyama K, *et al.* Characteristics of behavioral symptoms in right-sided predominant semantic dementia and their impact on caregiver burden: a cross-sectional study. *Alzheimers Res Ther* 2021; **13**: 166.

124 Shaw SR, El-Omar H, Ramanan S, *et al.* Anhedonia in Semantic Dementia-Exploring Right Hemispheric Contributions to the Loss of Pleasure. *Brain Sci* 2021; **11**: 998.

125 Ulugut Erkoyun H, van der Lee SJ, Nijmeijer B, *et al.* The Right Temporal Variant of Frontotemporal Dementia Is Not Genetically Sporadic: A Case Series. *J Alzheimers Dis JAD* 2021; **79**: 1195–201.

126 Ulugut H, Dijkstra AA, Scarioni M, *et al.* Right temporal variant frontotemporal dementia is pathologically heterogeneous: a case-series and a systematic review. *Acta Neuropathol Commun* 2021; **9**: 131.

127 Carnemolla SE, Kumfor F, Liang CT, Foxe D, Ahmed RM, Piguet O. Olfactory Bulb Integrity in Frontotemporal Dementia and Alzheimer’s Disease. *J Alzheimers Dis* 2022; **89**: 51–66.

128 Dong L, Wang J, Liu C, *et al.* Genetic Spectrum and Clinical Heterogeneity of Chinese Frontotemporal Dementia Patients: Data from PUMCH Dementia Cohort. *J Alzheimers Dis*; **89**: 893–901.

129 Koros C, Beratis I, Matsi S, *et al.* Prosopagnosia, Other Specific Cognitive Deficits, and Behavioral Symptoms: Comparison between Right Temporal and Behavioral Variant of Frontotemporal Dementia. *Vision* 2022; **6**: 75.

130 Paranhos T, Lucas T, de Salles A, Moll J, de Oliveira-Souza R. A presumptive association between obsessive compulsions and asymmetric temporal lobe atrophy: a case report. *J Med Case Reports* 2022; **16**: 21.

131 Pourriyahi H, Almasi-Dooghaee M, Imani A, Vahedi T, Zamani B. “Split-day syndrome”, a patient with frontotemporal dementia who lives two days in the span of one: a case report and review of articles. *Neurocase* 2022; **28**: 292–7.

132 Rossi G, Salvi E, Mehmeti E, *et al.* Semantic and right temporal variant of FTD: Next generation sequencing genetic analysis on a single-center cohort. *Front Aging Neurosci* 2022; **14**. DOI:10.3389/fnagi.2022.1085406.

133 Younes K, Borghesani V, Montembeault M, *et al.* Right temporal degeneration and socioemotional semantics: semantic behavioural variant frontotemporal dementia. *Brain* 2022; **145**: 4080–96.

134 Di Napoli J, Arighi A, Conte G, *et al.* Predominant right temporal lobe atrophy: Clinical, neuropsychological and structural differences based on amyloid status. *Eur J Neurol* 2024; **31**: e16124.

135 Frings L, Blazhenets G, Binder R, Bormann T, Hellwig S, Meyer PT. More extensive hypometabolism and higher mortality risk in patients with right- than left-predominant neurodegeneration of the anterior temporal lobe. *Alzheimers Res Ther* 2023; **15**: 11.

136 Geraudie A, Pressman PS, Pariente J, *et al.* Expressive Prosody in Patients With Focal Anterior Temporal Neurodegeneration. *Neurology* 2023; **101**: e825–35.

137 Gressie K, Kumfor F, Teng H, *et al.* Error profiles of facial emotion recognition in frontotemporal dementia and Alzheimer’s disease. *Int Psychogeriatr* 2023; : 1–10.

138 Hazelton JL, Devenney E, Ahmed R, *et al.* Hemispheric contributions toward interoception and emotion recognition in left-vs right-semantic dementia. *Neuropsychologia* 2023; **188**: 108628.

139 Hua AY, Roy ARK, Kosik EL, *et al.* Diminished baseline autonomic outflow in semantic dementia relates to left-lateralized insula atrophy. *NeuroImage Clin* 2023; **40**: 103522.

140 Kawakatsu S, Kobayashi R, Morioka D, *et al.* Clinicopathological diversity of semantic dementia: Comparisons of patients with early-onset versus late-onset, left-sided versus right-sided temporal atrophy, and TDP-type A versus type C pathology. *Neuropathology* 2023; **43**: 5–26.

141 Leocadi M, Canu E, Cividini C, *et al.* Brain structural abnormalities and cognitive changes in a patient with 17q21.31 microduplication and early onset dementia: a case report. *J Neurol* 2023; **270**: 1127–34.

142 Mesulam M-M, Gefen T, Flanagan M, *et al.* Frontotemporal Degeneration with Transactive Response DNA-Binding Protein Type C at the Anterior Temporal Lobe. *Ann Neurol* 2023; **94**: 1–12.

143 Ohm DT, Rhodes E, Bahena A, *et al.* Neuroanatomical and cellular degeneration associated with a social disorder characterized by new ritualistic belief systems in a TDP-C patient vs. a Pick patient. *Front Neurol* 2023; **14**. DOI:10.3389/fneur.2023.1245886.

144 Piccininni C, Marra C, Quaranta D, *et al.* Which components of famous people recognition are lateralized? A study of face, voice and name recognition disorders in patients with neoplastic or degenerative damage of the right or left anterior temporal lobes. *Neuropsychologia* 2023; **181**. DOI:10.1016/j.neuropsychologia.2023.108490.

145 Ramanan S, El-Omar H, Roquet D, *et al.* Mapping behavioural, cognitive and affective transdiagnostic dimensions in frontotemporal dementia. *Brain Commun* 2023; **5**: fcac344.

146 Ghirelli A, Spinelli EG, Canu E, *et al.* Clinical and neuroanatomical characterization of the semantic behavioral variant of frontotemporal dementia in a multicenter Italian cohort. *J Neurol* 2024; published online April 10. DOI:10.1007/s00415-024-12338-9.
