# Supplementary material for: Clinical recognition of frontotemporal dementia with right temporal predominance: a consensus statement from the International Working Group
Source: Commun Med (Lond). 2025 Dec 12;5:523. doi: 10.1038/s43856-025-01252-4 (PMC12700944; doi:10.1038/s43856-025-01252-4)
Supplement: Supplementary file 6 — Supplementary Data File 4 [file 43856_2025_1252_MOESM6_ESM.docx]

**Supplementary Table 4. Interpretation of the reported symptoms and consensus recommendations.**

| 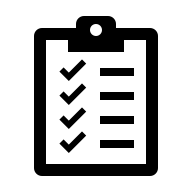 | **Real life examples** | **Published evidence** | **IWG Recommendations** |
| --- | --- | --- | --- |
| **B**  **L**  **M** | *“…She cannot recognize us anymore…”*  *“… He is struggling with naming famous actors while we are watching TV…”* | A considerable volume of research indicates that while visual face perception remains intact, there are significant impairments in identification, naming, familiarity, and semantic knowledge of individuals when tested using pictures (visual) or voice (auditory) stimuli. Notably, these capabilities are relatively preserved in the early stages of the condition when the information about individuals is presented through their names (verbal). | The term “**Knowledge loss for people via multimodal non-verbal stimuli**” provides a clear definition for clinicians and adequately explains the clinical presentation. Existing face-to-face tests are mainly available for Western, English-speaking populations. Cultural adaptation of those tests is essential. Additionally, current validated tools assess person identity via static pictures. Novel ecologically valid and culturally sensitive tools that combine visual and auditory information are warranted. |
| **B**  **L**  **M** | *“… As a bird expert, he can no longer identify birds from their calls…”*  *“…She had always been an expert on wild mushrooms and had never had any doubt about whether a species was edible or not. In the last year or so, she would pick mushrooms and toadstools indiscriminately, stating that they were all of the edible category; a blatantly wrong guess…”* | There is substantial evidence indicating category-specific semantic deficits (living versus non-living) when tested with visual and auditory stimuli. However, the number of studies and the sample sizes involved remain limited. | The term “**Knowledge loss for living beings via multimodal non-verbal stimuli**” provides a clear definition for clinicians. Better test designs and prospective multicultural studies are needed to elucidate whether the impairment is confined to socioemotionally relevant living organisms or extends to all living entities, and to assess if this symptom is an early marker of the syndrome. Currently, a few experimental tasks are available, but there is no validated tool for clinical use. During tool development/ validation, cultural sensitivities should be carefully considered. |
| **B**  **L**  **M** | *“… He drank from a bottle of soap, confusing it with food… He cannot differentiate the taste of soap or wine anyway…”*  *“… Little sense of smell, ignoring the strong smell of a skunk in their area…”*  *“… She cannot recognize many kitchen tools and food, she stopped cooking…”*  *“… He overreacts to innocuous physical stimuli…”* | There is substantial evidence indicating semantic deficits related to flavors, odors, sounds, and landmarks assessed through objective face-to-face tests. In contrast, studies on bodily sensations that report semantic losses rely primarily on interview methodologies. Other research on bodily sensations involving monitoring of the cardiac and autonomic systems presents inconsistent findings. It remains unclear whether these deficits are category-specific (affecting only modalities that trigger emotions or all modalities) and whether issues particularly with flavors and odors represent purely semantic deficits or are part of a broader deficit in hedonic valuation. | The term “**Knowledge loss for flavors, odors, sounds, landmarks, and bodily sensations via multimodal non-verbal stimuli**” provides a clear definition for clinicians. It is crucial to investigate whether these modalities manifest at early stages and whether the underlying issues are purely related to semantic deficits or involve other neural mechanisms such as altered hedonic valuation, particularly for chemical stimuli (flavors and odors). Additionally, the significance of internal monitoring in the understanding and evaluation of emotions needs clarification. Currently, a few experimental tasks are available, but there is no validated tool for clinical use. During tool development/ validation, cultural sensitivities should be carefully considered. |
| **B** | *“…He remarked, "There is water coming out of your eyes," when his wife was crying for the Virginia tragedy…”*  *“…She doesn't seem to be able to read emotions anymore…”* | Emotion recognition deficits have been extensively studied and validated by a large and diverse group of scientists | The term “**Knowledge loss for emotions via multimodal non-verbal stimuli**” provides a clear definition for clinicians and differs the syndrome from frontal system related impaired empathy process. It remains to be tested whether the bodily sensation of emotion dissipates before the cognitive recognition of the emotion, or vice versa. Existing face-to-face tests are mainly available for Western, English-speaking populations. Cultural adaptation of those tests is essential. Additionally, current validated tools assess emotion recognition via static pictures using only facial expressions. Novel ecologically valid tools that combine face, body posture and prosody are warranted. |
| **B** | *“…He has no recollection of either the events of September 11, 2001, or of the recent tsunami in Southeast Asia…”* | Substantial evidence from objective face-to-face tests indicates a loss of semantic knowledge about social events. Moreover, comparative studies show that the loss of knowledge for social information is more pronounced in patients with RATL neurodegeneration compared to those with LATL and frontal predominant atrophy, especially when the stimuli are multimodal and non-verbal. | The term “**Knowledge loss for social information**” provides a clear definition for clinicians. Existing face-to-face tests are mainly available for Western, English-speaking populations. During tool development/ validation, cultural sensitivities and ecological validity should be considered. |
| **B** | *“… He cannot read the body language and easily believe what people say… became more gullible…”* | Substantial evidence from objective face-to-face tests indicates deficits in comprehending paralinguistic cues. Moreover, comparative studies show that the loss of knowledge for paralinguistic cues is more pronounced in patients with RATL neurodegeneration compared to those with LATL and frontal predominant atrophy, especially when the stimuli are multimodal and non-verbal. | The term “**Knowledge loss for paralinguistic cues via multimodal non-verbal stimuli**” provides a clear definition for clinicians. Existing face-to-face tests are mainly available for Western, English-speaking populations. During tool development/ validation, cultural sensitivities and ecological validity should be considered. |
| **B** | *“… He has become very cold and does not show any emotions …”*  *“…She cries on seeing photos of grandchildren despite her lack of empathy with others …”* | Limited number of studies with limited sample sizes are available using objective measurements. Although they suggest that if an individual can no longer understand the semantic meaning of emotional information, the associated response appears compromised, it remains to be tested whether these altered emotional reactions are purely due to knowledge loss for emotions or whether alterations in physiological responses following neurodegeneration in RATL cause such symptoms | Given the lack of evidence elucidating the neural mechanisms, the IWG recommends the term **“Altered emotional expression,”** which is still relatively broad and does not fully reflect impaired neural domains. Yet it is more specific than current terms such as ‘lack of empathy’, and clearly describes the clinical outcome. It is crucial to investigate whether these modalities manifest at early stages and whether the underlying issues are purely related to semantic deficits or involve other neural mechanisms. Currently, a few experimental tasks are available, but there is no validated tool for clinical use. During tool development/ validation, cultural sensitivities and ecological validity should be considered. |
| **B** | *“…She has developed some loss of concern over personal boundaries, and is frequently bumping into people and staring almost inappropriately at strangers (to see if she recognizes them) …”* | This has been widely discussed in several theoretical models, suggesting that conceptual knowledge of social constructs and socially relevant cues are represented in the ATLs, and ATL related disinhibition is associated with loss of knowledge of social norms and expectations rather than a control, rule breaking or inhibition problem. However, no large sample size study has yet unveiled the neural components of altered social reaction using objective measurements | Given the lack of evidence elucidating the neural mechanisms, the IWG recommends the term **“Altered social reaction,”** which is still relatively broad and does not fully reflect impaired neural domains. Yet it is more specific and accurate than current terms such as ‘disinhibition’, and clearly describes the clinical outcome. It is crucial to investigate whether these modalities manifest at early stages and whether the underlying issues are purely related to semantic deficits or involve other neural mechanisms. Currently, no tasks are available for clinical use. During tool development, cultural sensitivities and ecological validity should be carefully considered. |
| **B** | *“… No more interest previous hobbies. She left all social clubs even if she was the president of the book club, and vice president of the gardening club. She prefers gardening by herself…”*  *“… Less enthusiastic for certain things such as meeting up friends, family members… instead, he decided to be the national golf champion and spent his entire time and money for this sport, even though he became extremely stingy regarding other daily life activities, including costs for showering…”* | Available studies using quantitative assessments are limited. One study employing the informant-based surveys highlights a unique role for right temporal lobe structures in modulating anhedonia. This study suggests that degeneration predominantly affecting right-hemisphere structures negatively impacts the ability to experience pleasure, resulting in diminished motivation. On the other hand, published clinical data, including the IWG dataset based on observations by clinicians and caregivers, indicate a marked shift from socially motivated activities to more solitary pursuits, for which individuals show increased motivation. Notably, no studies to date have employed objective, face-to-face tests to uncover the neural components underlying altered motivation and prioritization. | Given the lack of evidence elucidating the neural mechanisms, the IWG recommends the term **“Altered motivation for social interactions,”** which is still relatively broad and does not fully reflect impaired neural domains. Yet it is more specific than current terms such as ‘apathy’, and clearly describes the clinical outcome. Investigating whether these modalities manifest at early stages and identifying the underlying neural mechanisms is crucial. Beyond informant-based surveys for broad symptoms such as pleasure, apathy, motivation scales, more specific, objective, face-to-face tests targeting neural mechanisms are warranted. During tool development/ validation, cultural sensitivities and ecological validity should be carefully considered. |
| **B** | *“… She spends over eight hours each day writing and has recently published a book…”*  *“He plans his day and strictly follows his scheduled routine, which includes precise mealtimes, ensuring he walks 10,000 steps per day, listening to health podcasts, and rowing.”*  *“… he can spend his entire time with puzzles, computer games, cycling and writing religious quotes…”* | A large body of clinical studies, including the IWG dataset, based on observations by clinicians and caregivers indicates that patients spend considerable time and attention, exhibiting hyperfocus on certain activities and specific interests. However, there are no studies elucidating the neural underpinnings of hyperfocus on specific interests, nor are there objective tests available to assess this phenomenon. | Given the lack of evidence elucidating the neural mechanisms, the IWG recommends the term **“Hyperfocus on specific interests”** which is still relatively broad and does not fully reflect impaired neural domains. Yet it is more specific than current terms such as ‘mental rigidity, preoccupations, obsessions, compulsions, repetitive behavior’, and more comprehensive than single symptom-based terms such as ‘hyper-religiosity, hypergraphia etc.,’. Additionally, it clearly describes the clinical outcome. It is crucial to identify the underlying neural mechanisms. Objective, face-to-face tests targeting neural mechanisms are warranted. During tool development/ validation, cultural sensitivities and ecological validity should be carefully considered. |
| **B** | *“… Every Monday she dresses up all blue and eats only pasta…”*  *“…She had no musical background, but beginning two years ago, she acquired a strong preference for especially popular music and sometimes sang at home...”*  *“…He enjoys Chinese food now more than ever…”* | Alterations in personal preferences, such as food choices, colors, clothing, and aesthetic tastes, have been widely noted in the literature, including the IWG dataset. The current debate centers on whether these changes involve disturbances primarily in reward processing, shifts in hedonic values, or semantic loss as the underlying cause of strong personal preferences. There are no studies available using objective measurements to elucidate the neural mechanisms responsible for this clinical phenomenon. | Given the lack of evidence elucidating the neural mechanisms, the IWG recommends the term **“Altered hedonic valuation and personal preferences”** which is still relatively broad and does not fully reflect impaired neural domains. Yet it is more specific and accurate than current terms such as ‘mental rigidity, preoccupations, obsessions, compulsions, repetitive behavior’, and more comprehensive than single symptom-based terms such as ‘dietary changes, regimented diet, musicophilia etc.,’. Additionally, it clearly describes the clinical outcome. Investigating whether these modalities manifest at early stages and identifying the underlying neural mechanisms is crucial. Objective, face-to-face tests targeting neural mechanisms are warranted. During tool development/ validation, cultural sensitivities and ecological validity should be carefully considered. |

Strong scientific evidence; Substantial scientific evidence, better designs needed; Mild scientific evidence, mechanisms unknown;

**B**: Symptom can be reported as a behavioral problem by the caregiver **L**: Symptom can be reported as a language problem by the caregiver **M**: Symptom can be reported as a memory problem by the caregiver.
